# Identification and Validation of Compounds Targeting *Leishmania major* Leucyl-Aminopeptidase M17

**DOI:** 10.1021/acsinfecdis.4c00009

**Published:** 2024-05-16

**Authors:** Mirtha E. Aguado, Sandra Carvalho, Mario E. Valdés-Tresanco, De Lin, Norma Padilla-Mejia, Victoriano Corpas-Lopez, Martina Tesařová, Julius Lukeš, David Gray, Jorge González-Bacerio, Susan Wyllie, Mark C. Field

**Affiliations:** † Center for Protein Studies, Faculty of Biology, University of Havana, 10400 Havana, Cuba; ‡ Wellcome Centre for Anti-Infective Research, School of Life Sciences, University of Dundee, DD1 4HN Scotland, U.K.; § Centre for Molecular Simulations, University of Calgary, Calgary, AB T2N 1N4, Canada; ∥ Institute of Parasitology, Biology Centre, Czech Academy of Sciences, 37005 České Budějovice, Czech Republic; ⊥ Faculty of Sciences, University of South Bohemia, 37005 České Budějovice, Czech Republic

**Keywords:** drug discovery, *Leishmania*, M17 leucyl-aminopeptidase, RapidFire-MS, target validation

## Abstract

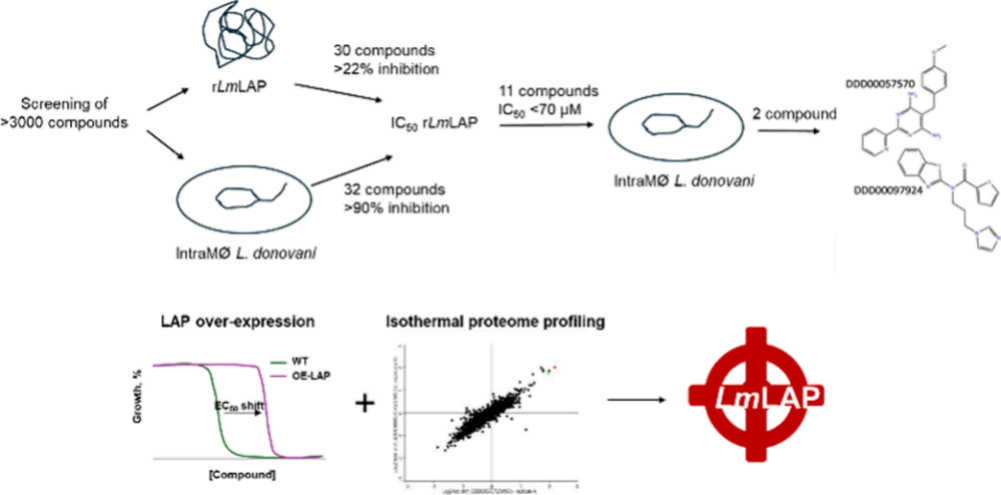

Leishmaniasis is a neglected tropical disease; there is currently no vaccine and treatment is reliant upon a handful of drugs suffering from multiple issues including toxicity and resistance. There is a critical need for development of new fit-for-purpose therapeutics, with reduced toxicity and targeting new mechanisms to overcome resistance. One enzyme meriting investigation as a potential drug target in *Leishmania* is M17 leucyl-aminopeptidase (LAP). Here, we aimed to chemically validate LAP as a drug target in *L. major* through identification of potent and selective inhibitors. Using RapidFire mass spectrometry, the compounds DDD00057570 and DDD00097924 were identified as selective inhibitors of recombinant *Leishmania major* LAP activity. Both compounds inhibited *in vitro* growth of *L. major* and *L. donovani* intracellular amastigotes, and overexpression of *Lm*LAP in *L. major* led to reduced susceptibility to DDD00057570 and DDD00097924, suggesting that these compounds specifically target *Lm*LAP. Thermal proteome profiling revealed that these inhibitors thermally stabilized two M17 LAPs, indicating that these compounds selectively bind to enzymes of this class. Additionally, the selectivity of the inhibitors to act on *Lm*LAP and not against the human ortholog was demonstrated, despite the high sequence similarities LAPs of this family share. Collectively, these data confirm *Lm*LAP as a promising therapeutic target for *Leishmania* spp. that can be selectively inhibited by drug-like small molecules.

Leishmaniasis denotes a spectrum of infectious diseases caused by multiple species of the genus *Leishmania*, with a broad range of symptoms and disease outcomes.^1^ An estimated ∼1 million new cases occur annually in ∼90 endemic countries, mainly in the tropics and subtropics, albeit that climate change is potentially increasing the geographic range of infections.^2^ Morbidities associated with leishmaniasis constitute an additional challenge to precarious socioeconomic situations in many of the affected developing countries.^3,4^

There are more than 20 species of *Leishmania*, with approximately 70 species of phlebotomine sandflies reported as transmission agents.^5,6^*Leishmania* species cause infections categorized as cutaneous, mucocutaneous, or visceral. Cutaneous leishmaniasis is the most common, resulting in skin lesions that are self-healing but can result in life-long scars. In mucocutaneous leishmaniasis, parasites disseminate from the site of infection to the mucous membranes and can cause large-scale tissue destruction. Complications, frequently from coinfection with bacteria or fungi, can be fatal. By contrast, visceral leishmaniasis (VL), commonly known as Kala-azar, is characterized by fever, hepatosplenomegaly and anemia. VL, the most aggressive and clinically important form of the disease, is caused exclusively by parasites of the *L. donovani* complex, i.e. *L. donovani, L. infantum* and *L. chagasi* in South America.^7,8^

There are currently no licensed vaccines for leishmaniasis and treatment, in its various forms, is dependent on a limited pool of drugs. All clinically used antileishmanials, including pentavalent antimonials, amphotericin B, paromomycin and miltefosine cause side effects and are threatened by emerging resistance.^9−11^ Thus, there is a pressing need for new drugs suitable for use in resource-poor settings.

Metallo-aminopeptidases are important for development and dissemination of *Leishmania* parasites within mammalian hosts and therefore attractive targets for new therapeutics.^12^ These enzymes function in the final stages of protein catabolism, in modulation of gene expression, antigen processing and immune defenses in various organisms.^13,14^ The leucyl-aminopeptidases (LAPs), belonging to the M17 family, are viable drug targets for important parasitic diseases, such as malaria.^15,16^ Using a compound specifically designed to inhibit *P. falciparum* M17-aminopeptidase (PfA-M17), the enzyme was demonstrated to be essential and druggable in *Plasmodium*. PfA-M17 also plays a role in providing malaria parasites with free amino acids for growth, mainly originating from digestion of hemoglobin.^15,17^

*L. major* LAPs (*Lm*LAPs) are classified in the TriTryp database according to their calculated isoelectric points as acidic (LmjF.11.0630; pI = 6.36), neutral (LmjF.33.2570; pI = 7.05) or basic (LmjF.23.0950; pI = 9.5), with the active sites and C-terminal domains highly conserved.^18^ The sequence identity percentages of the *Lm*LAP enzyme vary from 92% to 96% when compared to other LAPs within the same family. In contrast, the identity between *Lm*LAP and LAPs from *E. coli* and humans is approximately 30%.^19^ In a study by Timm et al. (2017), the LAP genes of trypanosomatids were examined, revealing a sequence identity of 48% between *Tc*LAP-A and *Lm*LAP-A. Furthermore, the percentage of identity between *Lm*LAP and LAPs from other organisms, such as bovine, human, *E. coli*, and Rickettsia, ranges from 41% to 46%.^20^

M17 LAPs have yet to be robustly validated as drug targets in *Leishmania*, but basic *Lm*LAP is expressed by all developmental stages and responsible for the vast majority of the cellular LAP activity.^19^ Since *Leishmania* parasites lack the capacity for synthesis of leucine and other branched-chain amino acids, it is likely that LAP plays a pivotal role in supplying these amino acids through hydrolysis of exogenous and endogenous proteins.^21^ In addition, leucine is a precursor for fatty acids and sterols biosynthesis.^22^

Basic *Lm*LAP can be inhibited by established LAP, aminopeptidase P and peptide-deformylase inhibitors,^19,23^ suggesting that this enzyme (*Lm*LAP from now) is potentially druggable and raising the possibility of selective inhibition in *Leishmania*. To test this hypothesis, we used RapidFire mass spectrometry (MS) to identify potent inhibitors of recombinant *Lm*LAP. Screening of ∼6,000 compounds from two diverse compound libraries identified two inhibitors with micromolar potency against r*Lm*LAP. These compounds specifically interact with M17 LAPs in *L. major* promastigotes, inhibit their growth and demonstrate little or no cytotoxicity against several mammalian cell lines, providing validation of LAP as a drug target for *L. major* and identification of compounds representing potential for future drug discovery.

## Results

### Optimization of RapidFire-MS for rLmLAP

**Figure 1:**
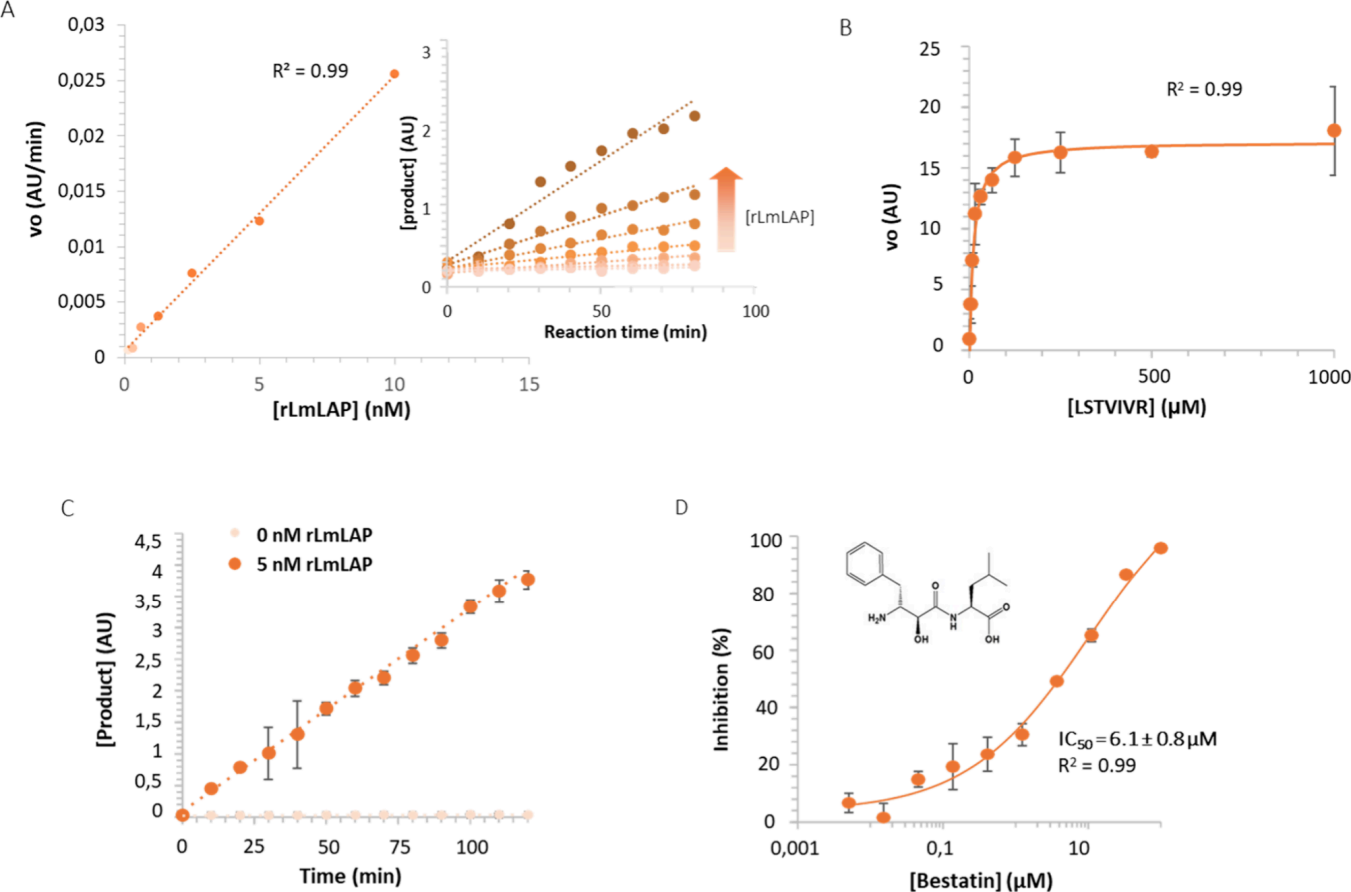
Optimization of RapidFire-MS for r*Lm*LAP with LSTVIVR. (A) Relationship between initial velocity (*v*_0_) and enzyme concentration. (B) Michaelis–Menten curve with LSTVIVR substrate within the range 0.19 μM to 1 mM. (C) Determination of the reaction interval in which the initial rate is maintained. (D) Concentration-dependent inhibition for the general LAP inhibitor bestatin.

To identify specific inhibitors of *Lm*LAP, we optimized a RapidFire-MS-based assay originally developed to identify inhibitors of *T. cruzi* LAP.^24,25^ Recombinant *Lm*LAP (r*Lm*LAP) was expressed in and purified from *Escherichia coli*.^23^ The peptide LSTVIVR was used as a substrate and the enzyme reaction product STVIVR was directly measured by RapidFire-MS, using STVIVR* as internal standard. To ensure that our r*Lm*LAP was enzymatically active, and we could detect the product of this reaction, we monitored the generation of product over time in assays using a fixed enzyme concentration (30 nM). Reassuringly, significant differences in product generation were observed between 0, 40, and 120 min reactions (data not shown). Then, the reaction was performed using four enzyme concentrations, ranging from 0.62 nM to 10 nM, at 2 mM of substrate for up to 80 min, to determine the linearity region. There was a linear relationship between all enzyme concentrations and the initial velocity (v_0_), and a linear increase in product generation observed up to 80 min (Figure 1A). An enzyme concentration of 5 nM was chosen for subsequent assays, since under these conditions the reaction product could be readily detected with sufficient signal-to-noise ratio.

The apparent K_M_ (appK_M_) of the peptide substrate at 5 nM r*Lm*LAP was determined to be ∼10 μM (Figure 1B) and this concentration was used in remaining experiments. The initial reaction velocity was constant for 120 min (Figure 1C). The linear relationship between v_0_ and enzyme concentration at 10 μM substrate was maintained for 120 min and, therefore, 60 min was the reaction time selected to perform the RapidFire-MS screen.

To test detection of r*Lm*LAP inhibition by RapidFire-MS we used the metallo-aminopeptidase inhibitor bestatin and demonstrated concentration-dependent inhibition of r*Lm*LAP with a half-maximum inhibitiory concentration (IC_50_) of 6.1 ± 0.8 μM (Figure 1D). To determine the robustness and the signal-to-noise ratio, a mock screen in the absence of inhibitors was performed. For this, two 384-well plates were used, with enzyme in all wells except the last column. The values of the Z’ robustness coefficient were 0.82 and 0.89 for each plate, and signal-to-noise ratios were 14.9 and 16.2, respectively (Table S1). These values are acceptable for high-throughput screens.

### High-Throughput Target Screening for Identification of rLmLAP Inhibitors

**Figure 2:**
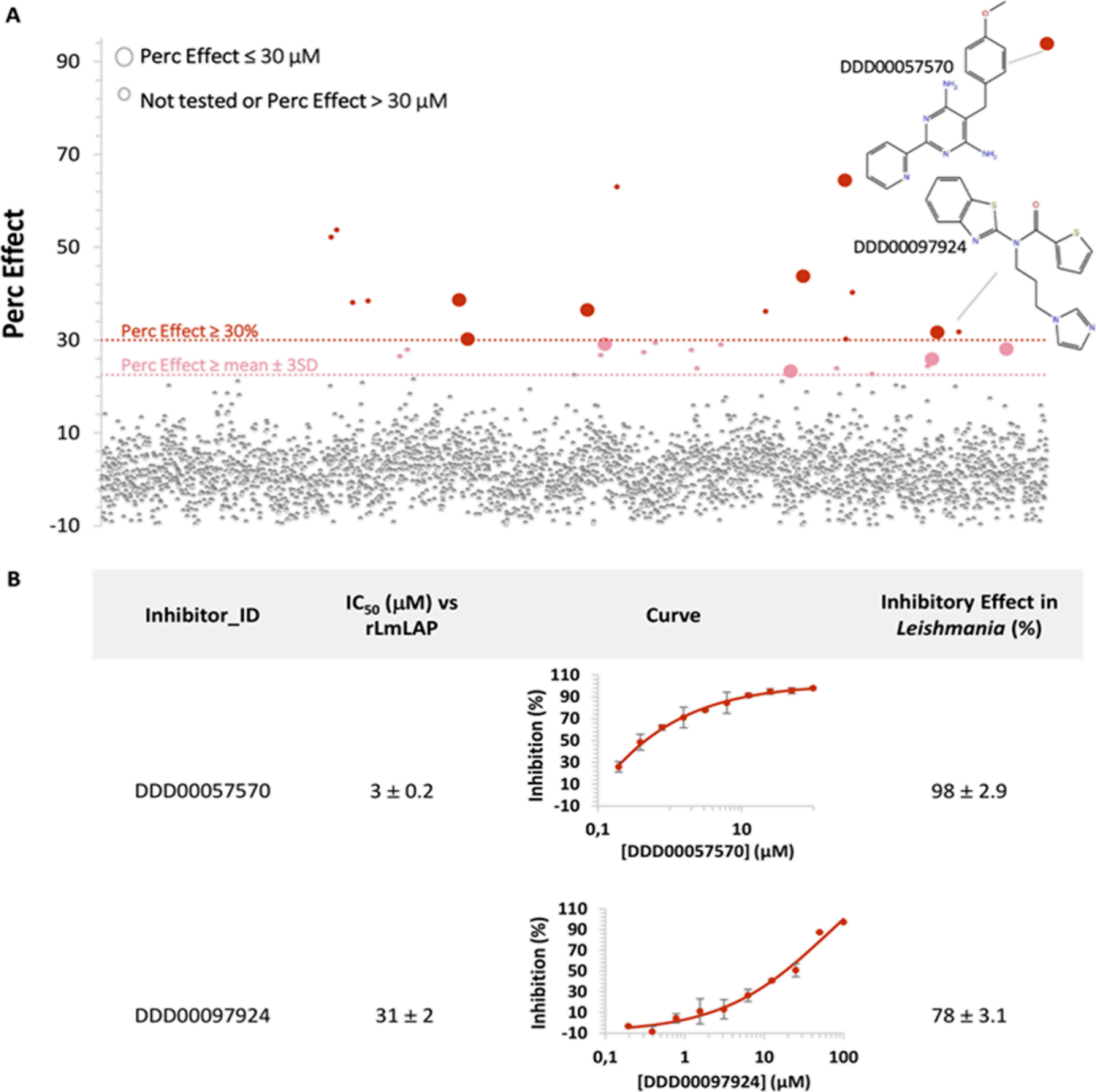
Compound library inhibition potency toward r*Lm*LAP, and single-point effect against *L. donovani* intracellular amastigotes. (A) Percentage of inhibition of r*Lm*LAP by the synthetic compounds. Compounds were tested at 30 μM by RapidFire-MS. The light-red dotted line indicates the hit selection threshold and dot color indicates the percentage effect; dark: ≥30%, light: ≥22%, while the two different sizes indicate the concentrations at which the referred effect is observed. The structures of the two selected compounds are shown. (B) IC_50_ values for r*Lm*LAP inhibition, concentration–response curves, and inhibition percentages at 50 μM on *L. donovani* amastigotes by the two selected compounds.

A library of 3,322 synthetic compounds with known protease inhibitor motifs was compiled and screened at 30 μM against r*Lm*LAP, using the standardized RapidFire-MS conditions. The compounds within the library maintain a diverse range of substituents of different sizes and hydrophobicity. We used a 30 μM screening concentration to enhance the chances of identifying compounds with weaker inhibition. Employing low stringency conditions is a common practice in screening protocols to avoid excluding potentially promising compounds. For hit selection, a threshold of 22% inhibition (mean ±3 SD) was imposed, resulting in identification of 30 compounds (Figure 2). These hits and a further 32 compounds (selected from another ∼3,000 compound library) demonstrating >90% inhibition of *L. donovani* viability in intramacrophage assays were selected for 10-point concentration–response studies. From these compounds, 11 demonstrated promising activities (IC_50_ values <70 μM). We selected compounds with an IC_50_ below 70 μM as a practical criterion. This approach allows to include a sufficient number of compounds for further evaluation, ensuring that promising results were not disregarded, while also keeping the number of compounds at a manageable level. One of them, DDD00057570, showed a potency that exceeded the established LAP inhibitor bestatin (3 ± 0.2 μM compared to 6 ± 0.9 μM, respectively) (Figure 2, Table 1).

**Table 1:**
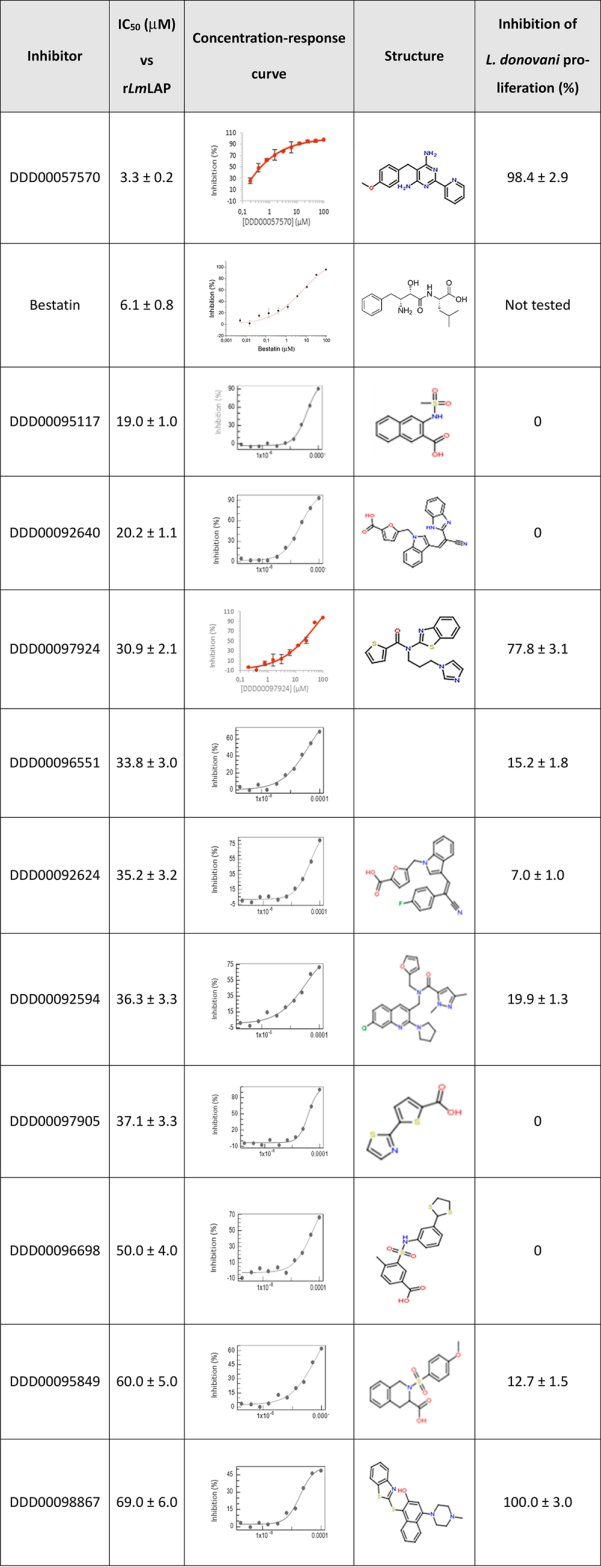
Concentration-Response Analysis for r*Lm*LAP and Single-Point Effect at 50 μM on *Leishmania donovani* Intracellular Amastigotes of Selected Compounds^a^

a Compounds were tested at ten concentrations in the 0.195 to 100 μM range, preincubating with enzyme (5 nM) for 20 min before LSTVIVR peptide substrate (10 μM) addition. IC_50_ values were calculated by nonlinear fit of the concentration–response function to the experimental data using OriginPro8 SR0 software. IC_50_ values are presented as the mean ± standard error (*n* = 3). Compounds are listed in decreasing order of r*Lm*LAP inhibition potency. The enzyme inhibitors were evaluated at 50 μM on *L. donovani* intracellular amastigotes and incubated with parasite-infected macrophages for 96 h. Parasite and host cells were stained with 5 μg/mL Hoechst 33342 and antileishmanial activity determined by imaging and counting of parasites. IC_50_: half-maximum inhibitory concentration.

The 11 compounds demonstrating the most promising activity against r*Lm*LAP were tested at a single concentration (50 μM) against *L. donovani* amastigotes in intramacrophage assays. *Lm*LAP and *Ld*LAP are 96.3/97.5% identical/similar respectively, with major differences located in loops distant from the active site. Therefore, it is highly likely that these compounds target the same enzyme in both species. Considering the indirect evidence and structural modeling in PyMOL, we expect similar drug sensitivity toward recombinant LAP in both *L. donovani* and *L. major*. Thus, measuring activity against *L. major* amastigotes is deemed unnecessary. The structural modeling reveals a close resemblance (RMSD < 0.1 Å) and supports nearly identical binding modes of the inhibitors to these enzymes, as the active site is well-conserved (results not shown). Remarkably, even at the residue side chain level, conservation is evident. Previous research has also demonstrated similar inhibitory effects of known aminopeptidase inhibitors on LAP activity in three Leishmania species.^19^ Only three of the 11 compounds tested inhibited parasite proliferation by >70% (Table 1). Notably, compound DDD00057570, the most potent r*Lm*LAP inhibitor, exhibited the second highest *in vitro* antileishmanial activity (98.4%), while compound DDD00097924, the fourth most potent inhibitor of r*Lm*LAP, exhibited the third highest *in vitro* antileishmanial activity (78%; Figure 2, Table 1). Two other compounds that inhibited the enzyme with IC_50_ ∼ 20 μM did not show inhibitory effects against parasites (DDD00095117 and DDD00092640). The case of the compound DDD00096698 with approximately 65% inhibition in the screening is a moderate inhibitor of the enzyme with an IC_50_ of 50 μM. However, it did not inhibit the proliferation of the parasite. Therefore, we did not consider them for further studies. Compound DDD00098867 had an antiparasitic activity of almost 100%, but this molecule was the weakest r*Lm*LAP inhibitor, with an IC_50_ ∼ 70 μM. Therefore, the *in vitro* antileishmanial activity of DDD00098867 may be due to its engagement with a molecular target other than LAP (Table 1). As our goal was to validate *Lm*LAP as a therapeutic target to treat Leishmaniasis, we decided to continue with compounds that showed inhibition of both enzymatic and parasite proliferation. The compounds DDD00057570 and DDD00097924 were selected for further studies.

### In Silico Modeling of rLmLAP:DDD00057570 and rLmLAP:DDD00097924 Complexes

**Figure 3:**
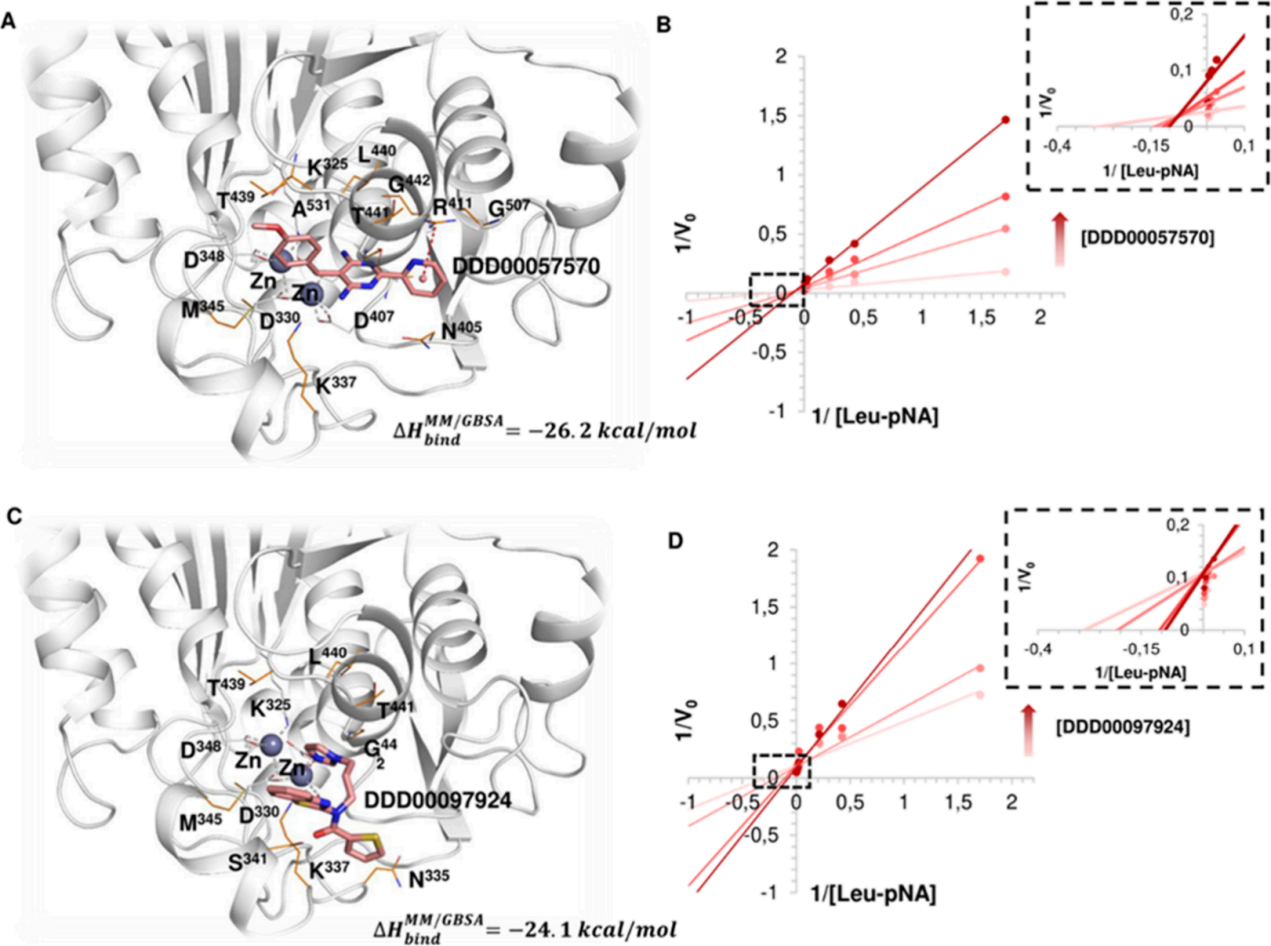
Kinetic and bioinformatic analyses of r*Lm*LAP:inhibitor interactions for DDD00057570 and DDD00097924. (A) Predicted binding mode of the r*Lm*LAP:DDD00057570 complex. For clarity, the interacting residues within 4 Å are represented as lines and van der Waals interactions between inhibitors and interacting residues are not indicated. Color codes: Inhibitor atoms: carbon in salmon, oxygen in red, nitrogen in blue and sulfur in yellow. LAP residues interacting with Zn^2+^ ions: carbon in gray, oxygen in red, and nitrogen in blue. LAP inhibitor’s interacting residues: carbon in orange, oxygen in red, nitrogen in blue and sulfur in yellow. The Zn^2+^ ions are represented as gray spheres. Zn^2+^ coordination is represented by gray dotted lines. (B) Lineweaver–Burk plot for diagnosis of the modality of r*Lm*LAP inhibition by DDD00057570. Data are reported as mean, *n* = 4, for 0, 15, 30, and 60 μM of the inhibitor in the presence of 300, 150, 75, 37.5, 4.7, 2.3, and 0.59 μM of the substrate (l-Leu-*p*NA). (C) Predicted binding mode of the r*Lm*LAP:DDD00097924 complex. Representation as described above. (D) Lineweaver–Burk plot for the diagnosis of the modality of r*Lm*LAP inhibition by DDD00097924 under the same conditions as for DDD00057570. Δ*H*_b_^MM/GBSA^: effective binding free energy by the MM/GBSA method.^27^

To better understand interactions between compounds DDD00057570 and DDD00097924 and *Lm*LAP, molecular modeling and docking studies were performed. Bestatin was used as a control and docking predicted a similar binding mode of this inhibitor to *Lm*LAP, in comparison with other LAPs (Figure S1). The models obtained with DDD00057570 and DDD00097924 indicate that the binding modes of each compound within the active site of *Lm*LAP are likely substantially different. First, DDD00057570 is predicted to establish van der Waals interactions with most residues lining the *Lm*LAP active site (K^337^, M^345^, N^405^, T^439^, L^440^, T^441^, G^442^, G^507^ and A^531^), including those coordinating the metal ions (K^325^, D^330^, D^348^, D^407^ and E^409^). These models suggest that DDD00057570 also establishes π-cation interactions between the most distal pyridine ring and residue R^411^ (Figure 3A). Notably, and contrary to characterized competitive inhibitors of LAP such as bestatin or actinonin,^19^ DDD00057570 does not establish polar interactions with any of the metal ions or coordinating residues. Indeed, our data indicate that DDD00057570 is a noncompetitive inhibitor (Figure 3B). In modeling, DDD00057570 partially occupies the active site of *Lm*LAP, which corroborates the noncompetitive inhibition mechanism with α > 1. DDD00057570 has binding affinity for both the free enzyme and the enzyme–substrate complex. In this case, α > 1, the inhibitor preferentially binds to the free enzyme.^26^

By contrast, DDD00097924 establishes van der Waals interactions with active site residues (N^335^, K^337^, S^341^, M^345^, T^439^, L^440^, T^441^ and G^442^), including those coordinating the Zn^2+^ cations (Figure 3C). However, the inhibitor coordinates a Zn^2+^ ion through the imidazole group, in a similar manner to actinonin.^18^ This model of binding is consistent with the competitive mode inhibition demonstrated for this compound (Figure 3D). The predicted poses for the inhibitors were further validated through binding free energy calculations using the MM/GBSA method.^27^ Essentially, a higher effective binding free energy is predicted for DDD00057570 (Δ*H*_b_^MM/GBSA^ = −26.2 kcal/mol) compared with that of DDD00097924 (Δ*H*_b_^MM/GBSA^ = −24.1 kcal/mol), in agreement with the lower IC_50_ observed for the first inhibitor (Figure 2). In addition, the calculated relative binding free energy (ΔΔ*G* = Δ*H*_b_^MM/GBSA^ [DDD00097924] - Δ*H*_b_^MM/GBSA^ [DDD00057570]) between these two compounds of 2.1 kcal/mol differs in less than 1 kcal/mol from the experimental ΔΔ*G* of 1.3 kcal/mol, validating the docking predictions.

### Selectivity of DDD00057570 and DDD00097924: rLmLAP vs Human LAP

Demonstrating selectivity is both critical and necessary for validation of a candidate drug/target combination. Given the significant similarities between the active sites of M17 aminopeptidases (e.g., 96% for *Lm*LAP-*L. donovani* LAP; 37% for *Lm*LAP- human LAP), it was vital to directly address the selectivity of DDD00057570 and DDD00097924 for inhibition of parasite over human LAP (*Hs*LAP). RapidFire-MS was optimized for recombinant human LAP (r*Hs*LAP) and determined that 150 nM r*Hs*LAP is within the linear region (Figure S2A) and that v_0_ kinetics are maintained for up to 180 min at this enzyme concentration (Figure S2B). The appK_M_ for the LSTVIVR peptide substrate with r*Hs*LAP is 600 μM (Figure S2C). These conditions were selected to test DDD00057570 and DDD00097924 against this human enzyme.

Both enzymes were assayed by RapidFire-MS at the conditions selected during optimization. IC_50_: half-maximum inhibitory concentration. Selectivity index: IC_50_(*Hs*LAP)/IC_50_ (*rLmLAP*). Two biological replicates were carried out with two technical replicates for each inhibitor.

IC_50_ values for both compounds against *Hs*LAP are reported in Table 2. Selectivity indices of DDD00097924 and DDD00057570 for r*Lm*LAP inhibition over r*Hs*LAP were calculated as 3 and >30-fold, respectively. At 100 μM, DDD00057570 inhibits r*Hs*LAP by 35% and r*Lm*LAP by 99%. The difference is not so marked for DDD00097924, which, at 100 μM, inhibited r*Hs*LAP and r*Lm*LAP activities by 62% and 75%, respectively (Table 2; Figure S3). Hence only DDD00057570 can be considered as specific toward the parasite enzyme.

**Table 2:** Selectivity of DDD00057570 and DDD00097924: r*Lm*LAP vs r*Hs*LAP

	IC_50_ values (μM)				
Inhibitor	r***Lm***LAP	r***Hs***LAP	Selectivity index	Inhibition (%) of r***Hs***LAP at 100 μM	Inhibition (%) of r***Lm***LAP at 100 μM
DDD00057570	3 ± 0.2	>100	>30	35	99
DDD00097924	31 ± 2	98	3	62	75

### Validation of LmLAP as the Target of DDD00057570 and DDD00097924

**Figure 4:**
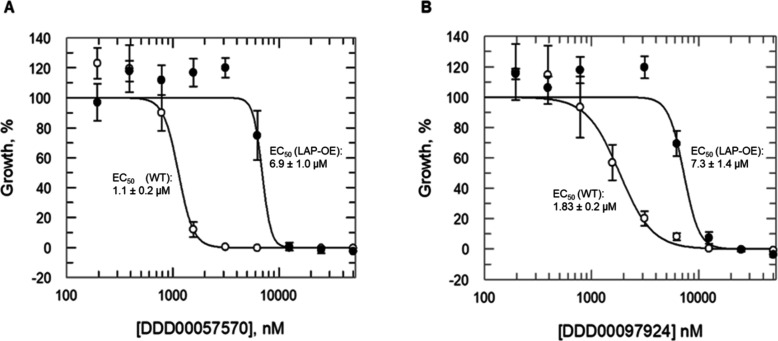
Impact of *Lm*LAP overexpression and drug susceptibility on *L. major* growth. The potency of compounds DDD00057570 (A) and DDD00097924 (B) was assessed against parental (WT, open circles) and *Lm*LAP-overexpressing (closed circles) *L. major* promastigotes. *L. major* representative proliferation curves for each compound are shown and the EC_50_ values corresponding to each specific curve shown in the figure. EC_50_ values (weighted means) of 2 ± 0.2 and 7 ± 1 μM were determined with DDD00057570 for parental and overexpressing cells, respectively; while values of 3 ± 0.2 and 11 ± 1 μM were determined with DDD00097924. Collated data from biological replicates are reported in Table S2.

To confirm *Lm*LAP as the molecular target of DDD00057570 and DDD00097924, and that its inhibition is responsible for the antileishmanial activity associated with both compounds, *L. major* promastigotes overexpressing *Lm*LAP were generated. Mass spectrometry confirmed that *Lm*LAP levels were ∼30-fold greater in the transgenic parasites compared with parental cells. Reassuringly, *Lm*LAP overexpression resulted in a notable ∼4-fold shift in half-maximum effective concentration (EC_50_) values for DDD00057570 and DDD00097924 between parental and overexpressing parasites (Figures 4A, 4B and S4), further confirming the interactions of DDD00057570 and DDD00097924 with *Lm*LAP.

### Isothermal Proteome Profiling (iTPP)

Thermal proteome profiling (TPP) is an orthogonal unbiased method to demonstrate compound-target engagement. The analysis is based on the principle that upon binding a drug leads to significantly altered thermal stability of a target protein. Here, we employed a rationalized version of our standard TPP protocol,^28^ known as isothermal proteome profiling (iTPP; 29), to assess if *Lm*LAP is stabilized by the presence of DDD00057570 or DDD00097924. Lysates of *L. major* promastigote were incubated in the presence of compound or diluent (DMSO) and subjected to a thermal shock at a single temperature (47 °C), rather than over a broad temperature range, as is the case for the canonical TPP protocol. Proteins that remain in solution following incubation at 47 °C were reduced, alkylated and digested with trypsin and LysC prior to derivatization with tandem mass tags (TMT). Pooled peptides were fractionated by high-pressure liquid chromatography (LC) and analyzed by LC–MS/MS prior to identification and quantitation. 2,618 proteins were identified, representing a proteome coverage of ∼31%, with quantification of 2,538 proteins.

**Figure 5:**
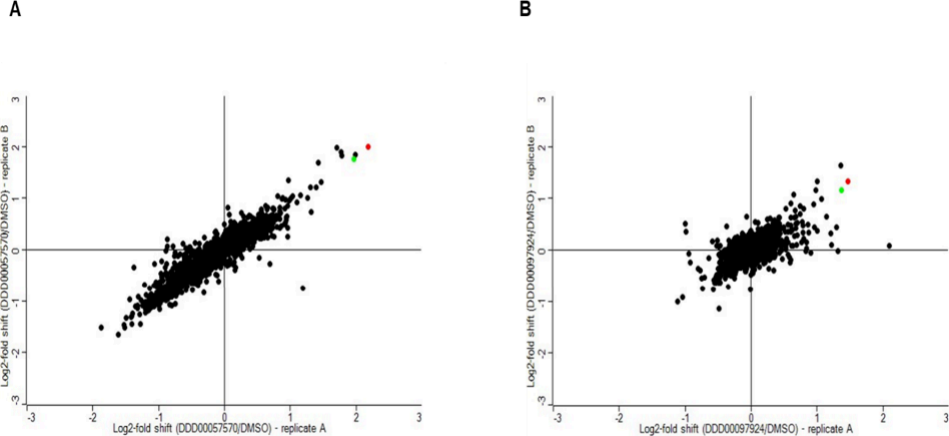
Target deconvolution utilizing iTPP. Plots show protein abundance log_2_ fold change between compound-treated and untreated lysates subjected to thermal shock at 47 °C. Lysates were exposed to DDD00057570 (A) and DDD00097924 (B). Data from biological replicates A and B are represented on the *x*- and *y*-axes, respectively. Two of the most highly stabilized proteins are shown in red (LmjF.11.0630) and green (LmjF.33.2570). The cutoff value for stabilization was log_2_-fold shift >1 in both replicates. See also Tables S3 and S4 for hit identification data.

Proteins whose relative abundance was increased in the presence of our putative LAP inhibitors, indicating enhanced thermal stability, were identified as hits. Four proteins were confirmed as thermally stabilized in the presence of DDD00057570 and three by DDD000097924. Two metallo-peptidases of the M17 family, acidic and neutral *L. major* LAPs LmjF.11.0630 (Q4QH17) and LmjF.33.2570 (Q4Q3T0), respectively, were top hits for both compounds (Figure 5). *Lm*LAP (the basic *L. major* LAP) suspected to be the primary target of both compounds was not identified in our data sets, likely due to low abundance or refractoriness to the MS analysis. However, it is notable that the two most stabilized proteins by both compounds are from the same enzyme class, namely M17 LAPs,^18^ further supporting direct interactions between DDD00057570 or DDD00097924 and M17 LAPs. Thus, these data support the hypothesis that both compounds elicit their leishmanicidal effects through combined inhibition of enzymes from this class.

### LAP Inhibitor Exposure and LmLAP Overexpression Elicits Morphological Changes

**Figure 6:**
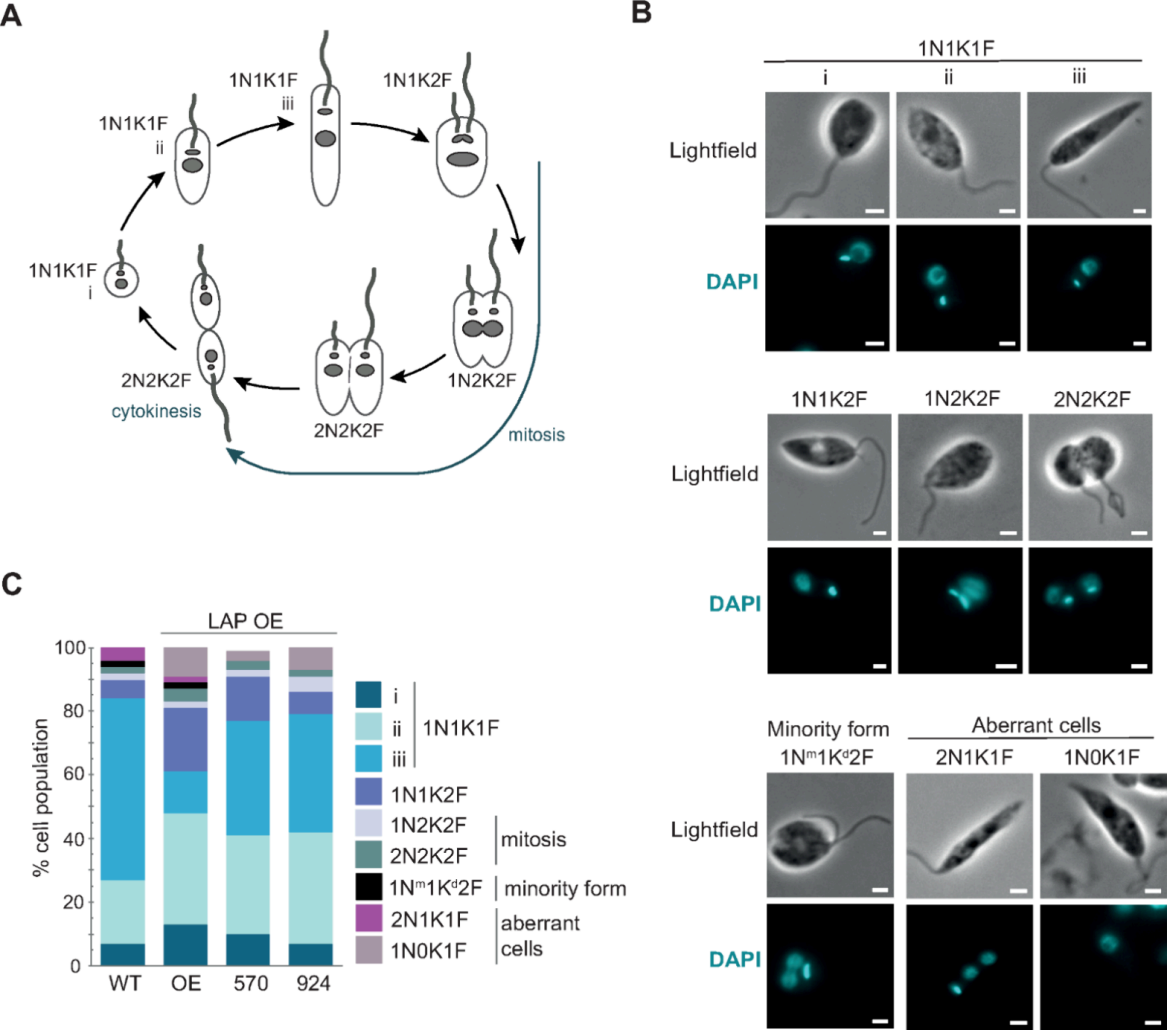
Cell cycle and morphology of *Lm*LAP-overexpressing cells. (A) Schematic of the *L. major* cell cycle. Number of flagella, nuclei and kinetoplasts in interphase and mitotic cells are shown (scheme is based on Ambit et al., 2011).^30^ (B) *L. major* cells across the cell cycle as depicted in (A). Images of light field and DAPI are shown for each stage of the cell cycle. Cells were fixed, DAPI-stained, and visualized by confocal microscopy. Scale bar: 2 μm. Cells with dividing nucleus and kinetoplast (1N^m^1K^d^2F) can be detected in normal *L. major* populations and are present in low percentages and have been described a minority configuration.^30^ Moreover, aberrant cells were observed, namely 2N1K2F cells and cells without a kinetoplast, 1N0K1F, the latter only occurring in *Lm*LAP OE cells. (C) Impact of DDD00057570 and DDD00097924 on the cell cycle. Cells were exposed to DDD00057570 and DDD00097924 at the corresponding EC_50_ concentrations for 72 h. *n* = 70 cells per condition. Cell cycle stages as in (A) and (B). N: nucleus. K: kinetoplast. F: flagellum. LAP: leucyl-aminopeptidase. WT: wild-type. OE: overexpressing. 570: DDD00057570. 924: DDD00097924. DAPI: 4′,6-diamidino-2-phenylindole.

To further explore the effects of compounds DDD00057570 and DDD00097924 on cells, *L. major* parental and *Lm*LAP overexpressors were exposed to both compounds and analyzed by confocal microscopy (Figure 6).^30^ Briefly, in *Leishmania* species, the cell cycle stage can be assessed from the number and position of the flagellum (F), kinetoplast (K, a network of mitochondrial DNA) and nucleus (N). In *L. major*, during interphase, a single kinetoplast, nucleus and flagellum are present (1N1K1F), while premitotic cells present two flagella (1N1K2F), with the growth of the second flagellum occurring very early during cell division. During mitosis, segregation of subcellular structures produces, first cells with two kinetoplasts (1N2K2F) and, subsequently, two nuclei (2N2K2F).^30^ Cells in cytokinesis present two fully segregated nuclei and kinetoplasts prior to cell division.^30^ These changes across the cell cycle are summarized in Figure 6A and B.

To determine if DDD00057570 and DDD00097924 impact the cell cycle, the number of cells in each developmental stage upon exposure to these compounds was assessed. This was important to determine since cells overexpressing *Lm*LAP had a significantly longer doubling time than parental cells (Figure S5). These findings suggest that the overexpression of *Lm*LAP induces cell cycle delay. The predominant morphology in the *L. major* wild-type population was the elongated 1N1K1F cells (stage iii, > 50% in the cell population; Figure 6A–C). However, overexpression of *Lm*LAP clearly affected morphology, as small 1N1K1F cells were predominant in the overexpressing population (stage ii, ∼35%). Moreover, the smallest, round interphasic 1N1K1F form (stage (i) and the 1N1K2F form were present at a higher frequency in the overexpressing cells as compared to parental cells (Figure 6C). When *Lm*LAP-overexpressing cells were exposed to DDD00057570 and DDD00097924, these morphological phenotypes were partially reversed and elongated 1K1N1F forms became predominant (∼36%), with concomitant reductions in the frequencies of the smallest, round cells and 1N1K2F cells (Figure 6B and 6C). Moreover, the population overexpressing *Lm*LAP accumulated aberrant cells without a kinetoplast (1N0K1F) (Figure 6C), which was reversed more efficiently by DDD00057570. Clearly, the overexpression of *Lm*LAP affected the normal progression of the cell cycle, which was reversed by both compounds. However, neither DDD00057570 nor DDD00097924 reversed the slower doubling time.

We confirmed that overexpression of LAP induces a series of subcellular abnormalities, including a predominant shape of round and swollen cells and swollen kinetoplast by electron microscopy (Figure S6). Abnormalities also include extra-large membrane-like compartments of variable size and morphology (Figure S6 panels E, F, H, I, J). An aggregation/agglomeration of small membrane-like compartments could be observed (Figure S6 panel E and H) or, alternatively, single, isolated compartments exhibited an extra-large volume inside the cell (Figure S6 panels F, I, J). Neither the composition nor identity of these membrane-bounded compartments is clear, but does occur as a response to the overexpression of *Lm*LAP, and supports the observation of cell cycle delay induced by *Lm*LAP overexpression. The addition of compounds reverses the shape of the swollen, round cells, partially restoring the shape of the elongated cells, and suggests that the compounds partly counteract the altered cell cycle and morphology of *Lm*LAP-overexpressing cells.

### Mammalian Cell Cytotoxicity of DDD00057570 and DDD00097924

**Figure 7:**
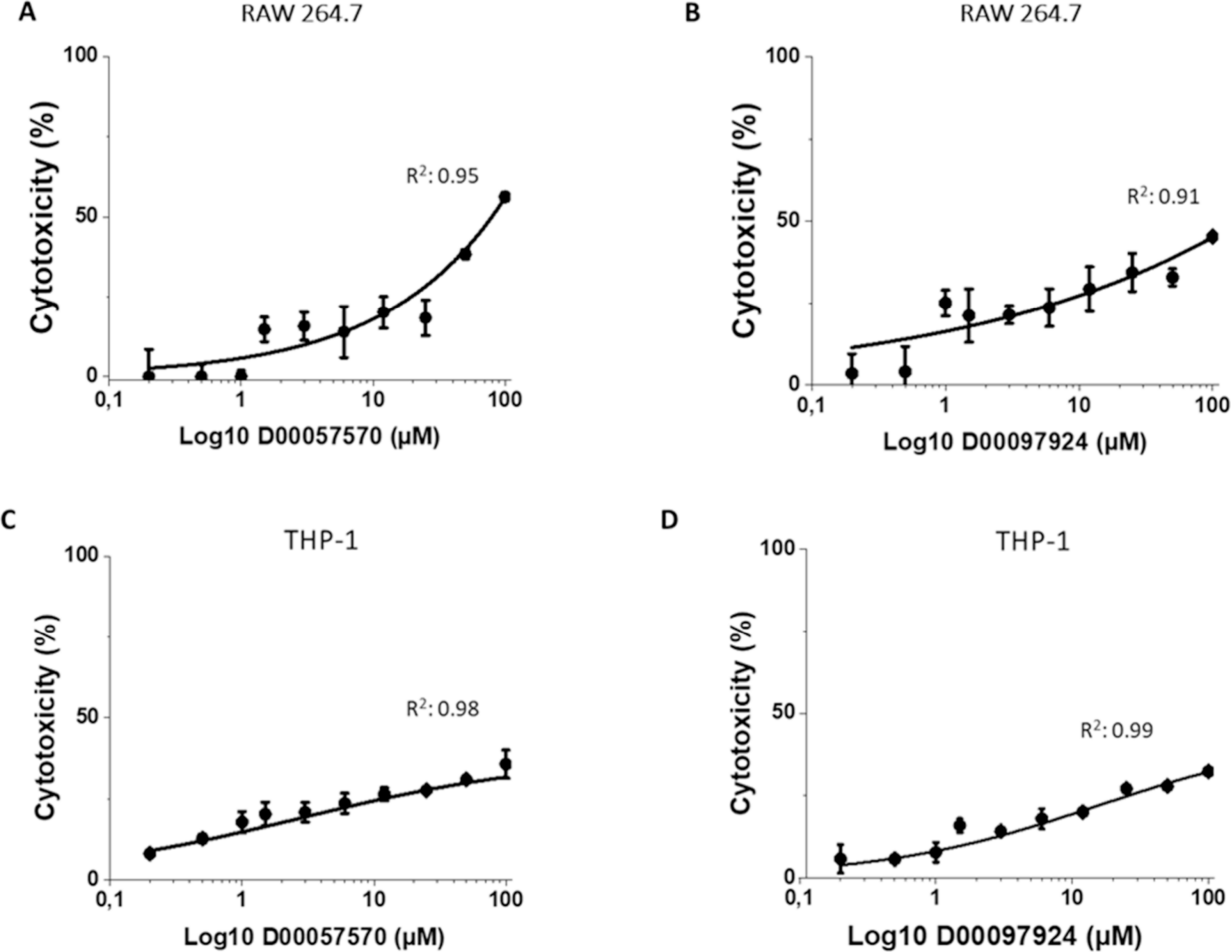
Cytotoxicity of DDD00057570 and DDD00097924 in mammalian cells. Compounds DDD00057570 (A and C) and DDD00097924 (B and D) were tested in murine RAW 264.7 (A and B) and human THP-1 macrophages (C and D). Cells were exposed to the compounds for 48 h and viability was determined with the MTT method. Data are presented as mean ± SD (*n* = 3).

The cytotoxicity of DDD00057570 and DDD00097924 against host cells was assessed by conducting a concentration–response study on two cell lines: RAW 264.7 murine macrophages and THP-1 human monocytes. Both compounds exhibit comparatively low cytotoxicity against these cell lines, as evidenced by half-maximum cytotoxic concentration (CC_50_) values >100 μM and absence of toxicity above the 50%-threshold at the highest concentration tested in all assays, with the exception of DDD00057570 in RAW 264.7 macrophages (Figure 7 and Table 2). The maximum inhibitory effect observed for DDD00057570 (100 μM) was 52% in assays with the RAW 264.7 macrophage cell line.

The compound DDD00057570, also tested at different concentrations against *L. donovani* intracellular amastigotes, has selectivity indices >13 regarding RAW 264.7 and THP-1 cells (Figure S7). These differences indicate that DDD00057570 prevents parasite growth by targeting a pathway specific to the pathogen and has not a general cytotoxic effect that also affects the host cells.

## Discussion

Metallo-aminopeptidases catalyze hydrolysis of amino acids from the N-termini of proteins or peptides. As they are implicated in pathogenesis in both bacteria and protists they are attractive targets for treatment of infectious diseases, although this potential has yet to be realized.^31,32,16^ M17 aminopeptidases are present in prokaryotes and eukaryotes and generally prefer N-terminal leucine and are thus often described as leucine-aminopeptidases (LAPs). LAPs are recognized as therapeutic targets in protists as inhibition of PfA-M17 is lethal to *P. falciparum* early in their life cycle.^15^ Moreover, PfA-M17 was recently validated both genetically and chemically as a potential drug target.^17^

In trypanosomatids LAPs play key roles in the infection of the host and participate in proliferation, differentiation, defense and dissemination, among others, although, with one exception, precise roles of LAPs remain uncharacterized.^12,16^ For example, the *T. cruzi* M17 LAP (LAPTc) is the major LAP activity in epimastigotes and inhibiting LAPTc is likely trypanocidal.^14,25^ In *T. brucei*, TbLAP1 (Tb927.8.3060, orthologous to LmjF.23.0950) could be involved in supplying leucine for sterol synthesis and, in consequence have roles in stress responses, signal transduction, and host cell invasion.^22,33,34^ TbLAP1 silencing delays cytokinesis through impact on kDNA replication.^35^ Additionally, *L. major* LAP-acid participates in protein catabolism, cell invasion and transcription regulation.^21,19,16^ PfA-M17 is essential for parasite development, since it is involved in host hemoglobin digestion.^15^ These studies highlight the diverse contributions M17 peptidases make to parasite biology, encompassing peptide catabolism, cellular processes and host interactions.

Significantly, all three *Leishmania* paralogs are shared with African trypanosomes and the majority of trypanosomatid flagellates. The subcellular location of each isoform is distinct. The *T. brucei* ortholog of LmjF.11.0630 is cytosolic and clearly essential by RNAi, while the LmjF33.2570 ortholog is nuclear and the *Lm*LAP ortholog was localized to the tripartite attachment complex (TAC)/kinetoplast DNA (kDNA) supercomplex and is essential.^36^ This suggests little redundancy between LAP paralogs. Despite being unable to detect *Lm*LAP by iTTP, the phenotype we observe from overexpression is highly similar to the failure to resolve the kDNA in TbLAP1-silenced trypanosomes and results in failure to complete cytokinesis. Overexpression of TbLAP1 in *T. brucei* (LmjF.23.0950 ortholog) is detrimental to growth, with alterations to the normal cell cycle and kinetoplast and mitochondrion replication.^35^ The cell cycle delay and the growth impairment induced by the overexpression of *Lm*LAP can be attributed to the involvement of M17 LAPs in meiosis and their ability to bind DNA. Furthermore, LAPs play a crucial role in metabolism, and altered expression can disrupt the balance of essential amino acids, such as leucine, this imbalance can impact energy production, nutrient utilization, and the biosynthesis of essential cellular components, ultimately affecting the efficiency of cell growth and division.

Hence, taken together we suggest that all three paralogs are likely impacted by DDD00057570 and DDD00097924. Multitargeting is potentially advantageous as it decreases the likelihood of resistance emerging, as all three isoforms would need to acquire relevant mutations for overall resistance to emerge.

Importantly both DDD00057570 and DDD00097924 exhibit a high selectivity for r*Lm*LAP against their human counterpart, which is an essential property for any compound under consideration for therapeutic use.^37^ Our *in silico* modeling predicts that *Lm*LAP has a hydrophobic and concave active site; these features suggest that the active site of *Lm*LAP is capable of establishing more favorable interactions with small hydrophobic ligands than *Hs*LAP, and that this may be the basis for selective action.^13,38^

Recently, there has been a significant increase in the progress for development of anti-Leishmania drugs, as well as consideration of new delivery mechanisms.^39−43^ These have included addressing new targets such as the proteasome and multiple proteases, together with characterization of new drug modalities, including benzoxaboroles. Further, nanoparticle delivery systems are being considered for improved activity of pre-existing compounds. These approaches are considering a wide range of targets and pharmacology, in part due to major advances in technology such as RapidFire-MS used here, deep sequencing and a concomitant ability to identify targets with increasing ease.^44^

The identification of potent and selective inhibitors for LAPs presents new possibilities for development of fit-for-purpose therapeutics against leishmaniasis. Further studies are required to investigate drug metabolism and pharmacokinetics (DMPK) of DDD00057570 and DDD00097924 and potential synergistic effects with existing drugs, as well as optimization by medicinal chemistry based on these scaffolds. Due to the clear conservation of the LAPs across trypanosomatids, the effect of both drugs can be explored in related flagellates, namely *T. cruzi* and *T. brucei*. In conclusion, the present study provides chemical validation of LAPs as viable drug targets in *Leishmania*. The identification of selective inhibitors and their demonstrated efficacy against *L. major* and *L. donovani* highlights the potential of targeting LAPs for the development of novel therapeutics against leishmaniasis

## Materials and Methods

### Production of Recombinant LmLAP

Recombinant *Lm*LAP (r*Lm*LAP) was expressed in *Escherichia coli* and purified precisely as previously described.^23^

### Aminopeptidase Activity Assay by RapidFire-MS

Assays were carried out as previously described,^24^ with some differences indicated below. The reaction mix consisted of 0 nM (control) and 30 nM r*Lm*LAP in 1× PBS, pH 7.0, 0.005% (v/v) NP-40, plus 7.5 μL of LSTVIVR substrate peptide in the same buffer. For product quantification, the peak areas of the LSTVIVR peptide were integrated with respect to the internal standard LSTVIVR* peptide. The C-terminal arginine was chosen because this amino acid is amenable to isotopic labeling.^45^

### Determination of the Linearity Region of the Initial Velocity as a Function of Enzyme Concentration

r*Lm*LAP was assayed at 0.62, 2.5, 5, and 10 nM with 2 mM (200× appK_M_) LSTVIVR substrate peptide. Different incubation times (0, 10, 20, 30, 40, 50, 60, 70, and 80 min) were tested for each enzyme concentration. The rest of the experimental conditions were maintained as previously described.

### Determination of *K*_M_ for rLmLAP

r*Lm*LAP was assayed at 5 nM with 10 concentrations of LSTVIVR peptide substrate, covering the range of 1.95–1000 μM. The rest of the experimental conditions were maintained as previously described.

### Determination of Incubation Time

r*Lm*LAP was assayed at 0 and 5 nM with 10 μM (1× appK_M_) of LSTVIVR substrate peptide, at 0, 10, 20, 30, 40, 50, 60, 70, 80, 90, 100, 110, and 120 min of reaction. Experimental conditions were maintained as previously described.

### Concentration–Response Study for Bestatin vs rLmLAP

r*Lm*LAP was assayed at 5 nM with 10 μM (1× appK_M_) of LSTVIVR substrate peptide. The enzyme was preincubated with bestatin or DMSO for 20 min. Bestatin was prepared at 10 concentrations in DMSO, covering the range from 0.39 to 100 μM. A control without inhibitor (same volume of DMSO, 0% inhibition) and a blank without enzyme and inhibitor (100% inhibition) were prepared. The remaining experimental conditions were maintained, as described above.

### Mock Screen with rLmLAP

To determine the robustness and the signal-to-noise ratio for the assay, a mock screen in the absence of inhibitors was performed. The enzyme was tested at 5 nM toward 10 μM (1 appK_M_) LSTVIVR peptide substrate. The reaction time was 60 min.

The robustness and the signal-to-noise ratio were calculated as follows:

Blank median = Median (Blank) (nonexcluded blank raw values)

High control (Ctrl) median = Median (Ctrl) (nonexcluded control raw values).
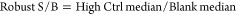
1
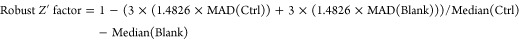
2where MAD: median absolute deviation.

### High-Throughput Screen for rLmLAP Inhibitors

A high-throughput screen was conducted against r*Lm*LAP using 3,383 compounds with known protease inhibitor motifs. The compounds were dispensed onto plates using an ECHO 550 acoustic dispenser (Labcyte, Sunnyvale, CA). The enzyme was assayed at 5 nM with 10 μM (1× appK_M_) LSTVIVR substrate for 60 min. Before adding the substrate, the enzyme was preincubated with 45 nL of the compounds or DMSO for 20 min. Compounds, dissolved in DMSO, were tested at 30 μM. A control without compound (same volume of DMSO, 0% inhibition) and a blank without enzyme and with compound (100% inhibition) were prepared. No replicates were performed for each inhibitor in this experiment. The remaining experimental conditions were kept constant as described above. The ActivityBase program from IDBS provider (https://www.idbs.com) was used to process and analyze the data. Selection criteria for potential inhibitors in this screen required an inhibition percentage greater than the mean (calculated from the inhibition percentages of all the inhibitors) plus three standard deviations (SD). All compounds were purchased commercially and are >95% pure by HPLC.

### Concentration–Response Study for Selected Compounds in the Inhibition of rLmLAP

To obtain IC_50_ data for screen-detected r*Lm*LAP inhibitors at a single concentration, ten-point concentration–response curves were generated for selected inhibitors showing greater than mean ±3 SD inhibition. The curves were prepared in 384-well plates with a maximum concentration of 100 μM and 1:2 serial dilutions in DMSO (0.195–100 μM) using an ECHO 550 acoustic dispenser. All other experimental conditions were as described above for RapidFire-MS optimization. Three replicates of each concentration of inhibitor were evaluated. The IC_50_ value was calculated by nonlinear fitting of a four-parameter logistic function to the experimental data. Curve fitting and IC_50_ calculations were performed using IDBS ActivityBase XE version 9.2.0.106. Inhibitory activity was determined using the peak area ratio, calculated as the reaction product (STVIVR) divided by its internal standard (*LSTVIVR). The peak area ratio of the reaction without enzyme was defined as 100% inhibitory activity, and that of the complete reaction mixture was defined as 0% inhibitory activity. At least three replicates were generated for each hit compound.

### Mode of Inhibition Studies for rLmLAP

The aminopeptidase enzymatic activity was determined by a continuous kinetic method.^26^ The reaction was performed in 50 mM sodium phosphate, pH 7.0, in the presence of 13.9 nM r*Lm*LAP (which is within the linear range of the v_0_ vs. enzyme concentration relationship) and 30 μM (1 appK_M_; 23) of the chromogenic substrate l-Leu-*p*-nitroanilide (Leu-*p*NA; 2 μL, in DMSO). The reaction was carried out at 25 °C in 96-well microplates (200 μL final volume), in a microplate spectrophotometer (FLUOstar OPTIMA, Germany). The increase of the absorbance at 405 nm (due to the release of the *p*NA chromogen) was recorded every 15 s for 5 min. Only the linear ranges of the typical curves, corresponding to substrate consumptions lower than 5% (v_0_ conditions) were used to measure the reaction velocity. Slopes with determination coefficients (R^2^) < 0.98 were not considered for linear fits.

For the determination of the inhibition mode, inhibitors were used at 0, 15, 30, and 60 μM. For each inhibitor concentration, the substrate Leu-*p*NA was added at different concentrations (0.59–300 μM). Experimental data were transformed, and the Lineweaver–Burk double reciprocal plots were constructed. The following equation was used:
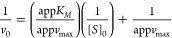
3where app*v*_max_ is the apparent maximal rate of the reaction, and [*S*]_0_ is the initial substrate concentration.^26^ The inhibition type was determined graphically from the lines of the double reciprocal plots.^26^

### Selectivity Study against Human LAP

To assess the inhibitory effect of DDD0005750 and DDD00097924 against the r*Hs*LAP enzyme, a similar experiment was performed as described in the concentration–response study for the r*Lm*LAP enzyme. r*Hs*LAP (LAP3 enzyme, Assay Genie, Ireland) was used at a concentration of 150 nM. The reaction mixture included 600 μM (1× appK_M_) LSTVIVR substrate peptide, 1 mM ZnCl_2_, and the reaction time was 180 min. The inhibitors were tested at concentrations ranging from 0.78 to 100 μM to determine inhibitory activity.

### In Silico Modeling of rLmLAP:DDD00057570 and rLmLAP:DDD00097924 Complexes

The three-dimensional (3D) structure of *Lm*LAP was predicted using ColabFold,^46^ combining the fast homology search of MMseqs2^47,46^ with AlphaFold2.^48^ Loops in the final model were further refined using the ModLoop^49,50^ online server. The final hexamer structure was assembled using the 3D structure of the acidic *L. major* LAP (PDB ID: 5NTH) as template.^18^ The coordinates of the ligand bound to acidic *L. major* LAP were translated to the predicted *Lm*LAP structure to guide the docking process (see below).

For molecular docking studies, bidimensional structures (2D) of the inhibitors in the Structure Data File (SDF) format were converted into 3D structures using Avogadro,^51^ and molecular docking simulations were performed with Autodock4Zn^52^ using the AMDock^53^ interface. Briefly, the docking input files were prepared with AutoDockTools scripts,^54^ while AutoGrid was employed to calculate the grid maps. Search space dimensions were determined dynamically as 2.9 times the radius of gyration of a docking compound. The grid center was set at the center of mass of the ligand bound in the active site, which was obtained from PDB (PDB ID: 5NTH). During the docking process, inhibitors were kept flexible within the grid box while the protein was held rigid. Default parameters were used for the remaining docking settings.

Energy minimization of predicted *Lm*LAP:inhibitor complexes were performed using GROMACS v 2022.3 software package,^55^ periodic boundary conditions (PBC), and the Amberff14SB force field.^56^ Ligand parameters were generated from the General Amber Force Field v2.11 (GAFF2),^57^ and charges calculated using AM1-BCC.^58,59^ All systems were neutralized (Na^+^/Cl^–^) and solvated with explicit water molecules, which were modeled by the TIP3P parameter set,^60^ in a cubic dodecahedron box. The distance between the protein–ligand complexes and the edge of the box was set to 10 Å. The hexameric *Lm*LAP:inhibitor systems consisted of ∼504,000 atoms with a periodic box of 172 Å × 172 Å × 172 Å (after solvation + ions).

AMBER-compatible parameters were used to describe the zinc center.^61^ This essentially avoided Zn^2+^ ions unbinding from the active site and/or incorrect pseudo valence bond formation (due to the use of a classical force field). The LINCS algorithm^62^ was used to constrain all the covalent bonds in nonwater molecules, while the SETTLE algorithm^63^ was used to constrain bond lengths and angles in water molecules. Energy minimization was carried out using the steepest descent algorithm followed by the conjugate gradient method. MM/GBSA was used to estimate the effective binding free energy of the predicted complexes. The Amberff14SB force field was used to calculate the internal term (Δ*E*_int_) as well as van der Waals (Δ*E*_vdW_) and electrostatic (Δ*E*_ele_) energies. The GB-Neck2 model^64^ (igb = 8) was used to estimate the polar component of the solvation energy (Δ*G*_GB_) using a high dielectric constant of 10 as recommended for highly charged binding sites.^65^

### *Leishmania donovani* Intracellular Assay

The effect of the compounds selected as inhibitors of r*Lm*LAP on the proliferation of *L. donovani* amastigotes was evaluated at a concentration of 50 μM. Macrophage cultures (ECACC, catalog number: 88081201) were used as host cells. Parasites were incubated with 4.27× 10^7^ macrophages, at a multiplicity of infection (MOI) of 5, at 37 °C with 5% CO_2_, overnight in DMEM medium supplemented with 4.5 g/L glucose and glutamine and 10% FBS, in a Millicell HY T-600 three-layer culture flask (Merck Millipore, Solna, Sweden). Extracellular parasites were removed by aspirating the cell culture supernatant and the macrophage monolayer was washed twice with 30 mL of DMEM medium without FBS. Macrophages were incubated for another 24 h in the above conditions with 133 μL of fresh DMEM medium prewarmed at 37 °C and supplemented with 10% FBS to obtain a high count of intracellular amastigotes.

Forty-two hours after macrophage infection, compounds (50 μM, 250 nL in DMSO) were dispensed using LabCyte ECHO into each well of 384-well dark flat bottom plates (Corning, New York, USA). The plates were incubated at 37 °C, 5% CO_2_, for 4 h (without compounds, with DMSO, to determine the initial level of infection) and for 96 h (with and without compounds). Subsequently, the cells were fixed with 30 μL of formaldehyde solution (11% formaldehyde and 0.15 mg/mL blue bromophenol in PBS) for 20–30 min, washed with PBS and stained with 5 μg/mL Hoechst 33342 DNA dye in PBS, 0.01% Triton X-100, and 0.05% thimerosal for 20 min.

### Concentration–Response Study in *L. donovani* Intracellular Amastigotes

Macrophages (2.5 × 10^7^, THP-1 cell line, ECACC: 88081201) were infected with *L. donovani* promastigotes (2 × 10^8^) overnight in RPMI medium supplemented with 10% FBS (GE Healthcare, UK) in a Millicell HY 3-layer cell culture flask T-600 (Merck Millipore, Sweden) at 37 °C in 5% CO_2_. Extracellular parasites were removed by aspirating the cell culture supernatant, and the promastigotes were washed three times with 500 μL of 1× PBS. Macrophages were further incubated in 500 μL of fresh warm RPMI medium with 10% FBS for an additional 24 h to achieve high intracellular counts of amastigotes. The cells were then washed, harvested by trypsinization, and dispensed at 5 × 10^4^ cells per well in 50 μL RPMI medium supplemented with 2% FBS into black flat-bottomed 24-well plates (Corning, USA) prestamped with drugs in DMSO, using an automated washer/dispenser EL406 (BioTek, USA) and liquid handling software (BioTek, USA).

Compounds (stock at 100 μM in DMSO) were dispensed using the LabCyte ECHO into each well of black flat-bottomed 24-well plates. Ten-point potency curves were generated (0.001–100 μM, 1:3 dilutions in DMSO), in technical duplicates. The total time between the start of infection and the start of compound exposure was approximately 36 h. Plates were then incubated at 37 °C in 5% CO_2_ for 4 h (without compound, with DMSO) and 96 h (with compound). The plates were subsequently fixed with 30 μL formaldehyde solution (11% formaldehyde and 0.15 mg/mL bromophenol blue in PBS) for 20–30 min, washed with PBS, and stained with 5 μg/mL Hoechst 33342 in PBS, 0.01% Triton X-100, and 0.05% thimerosal for 20 min. The concentration–response studies were performed at least three times in independent experiments.

For the two last experiments, images were taken with an INCELL 1000 microscope (Sigma-Aldrich, UK) and analyzed with the IN-Cell Developer Toolbox 1.8 program (Sigma-Aldrich, UK) to segment parasite nuclei and kinetoplasts, reporting the number of amastigotes per macrophage. Potencies of compounds against the parasite were calculated with the IDBS vendor ActivityBase software (Woking, UK, https://www.idbs.com) using the total number of amastigotes per well. All the data were transformed into percentage of inhibition, using DMSO as 0% control, in each plate. Experiments were performed using specific equipment and software, and the results were analyzed in triplicate to ensure accuracy.

### Cytotoxicity Assay

Cytotoxicity of DDD00057570 and DDD00097924 in mammalian cells was evaluated using murine leukemia RAW 264.7 macrophages (ATCC TIB-71) and human THP-1 monocytes (ECACC 88081201). The cell lines were cultivated in RPMI-1640 medium supplemented with 10% heat-inactivated FBS, 0.15% (w/v) NaHCO_3_, 100 units/mL penicillin, and 100 mg/mL streptomycin at 37 °C in a humidified atmosphere containing 5% CO_2_. The human cell line THP-1 was differentiated into adherent macrophages before addition of the test compounds. Differentiation was maintained for 72 h at 37 °C and 5% CO_2_^66,67^ in RPMI medium supplemented with 20% FBS medium, 2 mM glutamine and 8 nM phorbol-12-myristate-13-acetate (PMA).

RAW and THP-1 macrophages at 1.0 × 10^5^ cells/well in 100 μL of RPMI medium supplemented with 10% FBS were seeded in 96-well plates. Ten-point potency curves were generated in technical replicates in the range of 0.001–100 μM, with plates incubated at 37 °C in 5% CO_2_ for 48 h (with compounds and the control with DMSO). Blank contains only culture medium. After 48 h, the cell viability was determined by the MTT colorimetric assay. The cells were incubated in the presence of 10 μL of MTT (3-(4,5-dimethylthiazol-2-yl)-2,5-diphenyl tetrazolium bromide) dye solution for 4 h at 37 °C in 5% CO_2_ in the absence of light. Formazan crystals were, then, dissolved by addition of 100 μL 10% sodium dodecyl sulfate to each well. The absorbance signal was measured in a Spectra Max microplate reader (Molecular Devices) at λ_abs_ 595 nm and λ_abs_ 690 nm. IC_50_ values were determined by fitting a sigmoidal concentration–response curve to the data using GraphPad Prism v.8. Each assay was developed in triplicate. The relative cell viability (%) compared with control cells (exponential-phase cells not submitted to treatment) was calculated as follows:
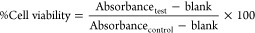
4

### Calculation of the Selectivity Index

The cell selectivity index (SI) was calculated as follows:
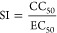
5where CC_50_ is the concentration able of killing 50% of mammalian cells and EC_50_ is the effective concentration that kills 50% of the intracellular parasites.

### Target Validation

#### *L. major* Promastigote Culture

*L. major* promastigotes were cultured in HOMEM (Gibco) supplemented with 10% heat inactivated FBS at 28 °C.

#### Generation of LmLAP-Overexpressing *L. major* Promastigotes

The basic *L. major* cytosolic leucyl aminopeptidase gene (LmjF.23.0950) was synthesized (GeneArt, Invitrogen) and cloned into the overexpression vector pIRT1_SAT by using *Bgl*II restriction sites. The pIRT1_SAT_LmLAP construct was sequenced to confirm correct sequence. Mid log phase *L. major* cells (1 × 10^7^ cells) were transfected with 10 μg of heat-sterilized DNA using the Human T-Cell Nucleofector kit and AMAXA Nucleofector electroporator (program V-033). After a 24 h recovery period, nourseothricin was added to cultures to a final concentration of 100 μg/mL.

#### Validation of LmLAP Overexpression

*L. major* cell lysates were made as previously described.^68^ Briefly, mid log phase promastigotes were harvested by centrifugation (1912*g*, 15 min, 4 °C) and washed twice with ice-cold PBS. Cell pellets were resuspended in 1.5 mL of ice-cold lysis buffer (1 mM EDTA, 1 mM DTT, 100 μM TLCK, and 1× Roche EDTA-free complete protease inhibitor cocktail in 50 mM potassium phosphate buffer, pH 7.4) and biologically inactivated by 3 freeze–thaw cycles in a dry ice/ethanol bath. Cell disruption was performed at 30 kpsi (Constant Systems, UK) and lysates cleared by centrifugation (20,000*g*, 10 min, 4 °C). The protein concentration of the supernatant was determined using the Bio-Rad Protein Assay. LC-MS/MS analysis was performed as described by Paradela et al., 2021.

#### Drug Sensitivity Assays

*L. major* promastigotes were seeded in 96-well plates at 5 × 10^4^/mL, exposed to 2-fold serial dilutions of test compounds, and incubated at 28 °C for 72 h. Resazurin was, then, added to each well to a final concentration of 50 μM, and fluorescence was measured after 2–3 h (excitation of 528 nm and emission of 590 nm). The EC_50_ was calculated by processing the collected data by GRAFIT (version 5.0.4, Erithacus Software) and fitting them to the following two-parameter equation:
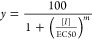
6where [*I*] is the inhibitor concentration, and *m* is the slope. Experiments were performed on at least three independent biological replicates. Data are presented as the weighted mean ± standard deviation.

#### Target Engagement Analysis by Isothermal TPP

Cell lysates were obtained as detailed above. Isothermal TPP assays were performed as previously described^29^ but here lysates were incubated at two temperatures: 28 and 47 °C. The first temperature (28 °C) acts as a control temperature at which the protein levels are not altered by the presence of the test compound. The second temperature (47 °C) is the *Leishmania* spp. median *T*_m_ (melting temperature or temperature 50%) according to our interim data. Briefly, cell lysates were adjusted to 1 mg/mL and were incubated in the presence of the drug (concentration equivalent to 10× EC_50_) or diluent (DMSO) for 30 min. These samples were aliquoted and submitted to a temperature shock at the designated temperature for 3 min, then 3 min at room temperature, and finally 3 min on ice. The soluble part was isolated by ultracentrifugation (100,000*g*, 20 min, 4 °C).

### Sample Processing and LC-MS/MS Analysis

All aspects of sample processing, TMT-labeling, fractionation by HPLC into 10 fractions, LC-MS/MS, and protein identification and quantitation were described previously.^28,68^ Proteins with less than three peptides and those with increased stability at 28 °C were disregarded.

Proteins were identified by searching the MS and MS/MS data for the peptides against *L. major* strain Friedlin (TriTryp version 61, tritrypdb.org) using MaxQuant (http://maxquant.org/, version 2.1.4.0). All proteomics data sets have been deposited to the ProteomeXchange Consortium via the PRIDE^69^ partner repository with the identifier PXD00000. Data were analyzed with Perseus v 1.6.15.^70^ The reporter intensities of each protein were extracted and normalized to the control temperature reporter intensity (28 °C), then these normalized values were used to calculate the log_2_ transformed fold-changes (FC) of DMSO versus drug-treated samples. Proteins stabilized (log_2_ FC > 1 in both replicates) were selected as putative hits.

### Microscopy Assays

*L. major* promastigotes (WT and *Lm*LAP OE) were grown in supplemented HOMEM media as described above, in T25 flasks. *Lm*LAP OE cells were grown in the presence of DDD00057570 or DDD00097924 at the EC_50_ or in the absence of inhibitors (control) for 72 h. *L. major* WT cells were grown at control conditions. Briefly, cells were fixed directly in culture media with 3% (v/v) paraformaldehyde for 15 min at room temperature, washed three times with excess D-PBS, permeabilized with 0.2% Triton X-100 (v/v) in PBS for 5 min, and washed three times in excess PBS. Slides were mounted with mounting medium with DAPI (Vectashield Lab H-1000–10). Microscopy was carried out on a Zeiss microscope and images captured using software Zen Blue (Zeiss). Analysis of images and figure preparation were made in Omero.^71^

### Electron Microscopy

*L. major* promastigotes (WT and LAP OE) were grown as described above. LAP OE cells were grown at different conditions; (A) control conditions (no compounds added), (B) DDD00057570 and (C) DDD00097924 at EC_50_. Cells were exposed to the compounds for 72 h. Cells were centrifuged at 590 rpm and then resuspended in fixation solution (2.5% glutaraldehyde in PBS) and kept at 4 °C. Postfixation was performed with 2% osmium tetroxide for 2 h at room temperature. Dehydration was carried out using series of acetone (30, 50, 70, 80, 90 and 100%). Samples were infiltrated into EPON resin, using three different mixtures of resin:acetone (1:2, 1:1 and 2:1), each left for 1 h. Embedding into pure resin EPON and polymerization were carried out at 62 °C for 48 h. Ultrathin sections (90 nm) were cut on copper grids and contrasted using uranyl acetate and lead citrate. Sections were observed using a Jeol 1400 Flash transmission electron microscope at 120 kV.

### Assessment of the Effect of Compounds on Parasite Growth

To assess the effect of the inhibitors on the cumulative growth of the parasites, WT and LAP OE cells were seeded at 1 × 10^6^/mL in the absence of compounds (control), with 0.1% DMSO (compound solvent) or in the presence of DDD00057570 or DDD00097924 at the corresponding EC_50_. The densities of the cultures were registered daily, and the cultures dilutes back to 1 × 10^6^/mL. The DMSO concentration 0.1% corresponds to the maximum used when compounds were added to cultures in this experiment. The antileishmanial assay was performed with resazurin as described above.

### Statistical Analysis

All experiments were performed at least three times, and the data are presented as the mean ± standard deviation (SD).

## Supporting Information Available

Additional experimental data, tables and figures. Table S1: Zn′ robustness coefficients and signal-to-noise ratios of mock screen for r*Lm*LAP. Table S2: Impact of *Lm*LAP overexpression on drug sensitivity. Table S3: iTPP hits stabilized in the presence of DDD00057570. Table S4: iTPP hits stabilized in the presence of DDD00097924. Figure S1: Bioinformatics analyses of rLmLAP:bestatin interactions using PyMol. Figure S2: Optimization of RapidFire-MS assay for *Hs*LAP. Figure S3: Dose–response for inhibition of *Hs*LAP by DDD00057570 and DDD00097924. Figure S4: Confirmation of *Lm*LAP overexpression in transgenic promastigotes by TMT quantitation. Figure S5: Cumulative growth of WT and LAP OE cells. Figure S6: Ultrastructure morphology of *L. major* promastigotes after treatment with compounds DDD00057570 and DDD00097924. Figure S7: EC_50_ values for DDD00057570 against *Leishmania donovani* intracellular amastigotes (PDF)

## References

[ref1] Burza S.; Croft S. L.; Boelaert M. Leishmaniasis. Lancet. 2018, 392 (10151), 951–970. 10.1016/S0140-6736(18)31204-2. 30126638

[ref2] World Health Organization. Leishmaniasis. 2023, Retrieved from https://apps.who.int/neglected_diseases/ntddata/leishmaniasis/leishmaniasis.html.

[ref3] Desjeux P. The increase in risk factors for leishmaniasis worldwide. Transactions of the Royal Society of Tropical Medicine and Hygiene. 2001, 95 (3), 239–243. 10.1016/S0035-9203(01)90223-8. 11490989

[ref4] World Health Organization. Leishmaniasis. 2023, Retrieved from https://www.who.int/news-room/fact-sheets/detail/leishmaniasis.

[ref5] Boelaert M.; Sundar S. Leishmaniasis. In Manson’s Tropical Diseases, 23rd ed.; Farrar J.; Hotez P. J.; Junghanss T.; Kang G.; Lalloo D.; White N., Eds.; Elsevier Inc.: Philadelphia, 2014; pp 631–651.http://lib.itg.be/pdf/itg/2013/2013mtdi0631.pdf.

[ref6] Mann S.; Frasca K.; Scherrer S.; Henao-Martínez A. F.; Newman S.; Ramanan P.; Suarez J. A. A Review of Leishmaniasis: Current Knowledge and Future Directions. Curr. Trop Med. Rep. 2021, 8 (2), 121–132. 10.1007/s40475-021-00232-7. 33747716 PMC7966913

[ref7] Lupi O.; Bartlett B. L.; Haugen R. N.; Dy L. C.; Sethi A.; Klaus S. N.; Machado Pinto J.; Bravo F.; Tyring S. K. Tropical dermatology: Tropical diseases caused by protozoa. J. Am. Acad. Dermatol. 2009, 60 (6), 897–925. quiz 926–928 10.1016/j.jaad.2009.03.004. 19467364

[ref8] World Health Organization. Neglected tropical diseases. 2021, Retrieved from https://www.who.int/health-topics/neglected-tropical-diseases/#tab=tab_1.

[ref9] Alvar J.; Vélez I. D.; Bern C.; Herrero M.; Desjeux P.; Cano J.; Jannin J.; den Boer M. WHO leishmaniasis control team Leishmaniasis worldwide and global estimates of its incidence. PLoS One 2012, 7 (5), e35671 10.1371/journal.pone.0035671. 22693548 PMC3365071

[ref10] Mohapatra S. Drug resistance in leishmaniasis: Newer developments. Trop Parasitol. 2014, 4 (1), 4–9. 10.4103/2229-5070.129142. 24754020 PMC3992802

[ref11] Kedzierski L.; Sakthianandeswaren A.; Curtis J. M.; Andrews P. C.; Junk P. C.; Kedzierska K. (2009) Leishmaniasis: current treatment and prospects for new drugs and vaccines. Curr. Med. Chem. 2009, 16 (5), 599–614. 10.2174/092986709787458489. 19199925

[ref12] De Almeida Nogueira N. P.; Morgado-Díaz J. A.; Menna-Barreto R. F.; Paes M. C.; da Silva-López R. E. Effects of a marine serine protease inhibitor on viability and morphology of Trypanosoma cruzi, the agent of Chagas disease. Acta Trop. 2013, 128 (1), 27–35. 10.1016/j.actatropica.2013.05.013. 23770204

[ref13] Matsui M.; Fowler J. H.; Walling L. L. Leucine aminopeptidases: diversity in structure and function. Biol. Chem. 2006, 387 (12), 1535–1544. 10.1515/BC.2006.191. 17132098

[ref14] Cadavid-Restrepo G.; Gastardelo T. S.; Faudry E.; de Almeida H.; Bastos I. M.; Negreiros R. S.; Lima M. M.; Assumpção T. C.; Almeida K. C.; Ragno M.; Ebel C.; Ribeiro B. M.; Felix C. R.; Santana J. M. The major leucyl aminopeptidase of Trypanosoma cruzi (LAPTc) assembles into a homohexamer and belongs to the M17 family of metallopeptidases. BMC Biochem. 2011, 12, 46 10.1186/1471-2091-12-46. 21861921 PMC3179936

[ref15] Harbut M. B.; Velmourougane G.; Dalal S.; Reiss G.; Whisstock J. C.; Onder O.; Brisson D.; McGowan S.; Klemba M.; Greenbaum D. C. Bestatin-based chemical biology strategy reveals distinct roles for malaria M1- and M17-family aminopeptidases. Proc. Natl. Acad. Sci. U. S. A. 2011, 108 (34), E526 10.1073/pnas.1105601108. 21844374 PMC3161592

[ref16] Aguado M. E.; Izquierdo M.; González-Matos M.; Varela A. C.; Méndez Y.; Del Rivero M. A.; Rivera D. G.; González-Bacerio J. Parasite Metalo-aminopeptidases as Targets in Human Infectious Diseases. Curr. Drug Targets. 2023, 24 (5), 416–461. 10.2174/1389450124666230224140724. 36825701

[ref17] Edgar R. C. S.; Siddiqui G.; Hjerrild K.; Malcolm T. R.; Vinh N. B.; Webb C. T.; Holmes C.; MacRaild C. A.; Chernih H. C.; Suen W. W.; Counihan N. A.; Creek D. J.; Scammells P. J.; McGowan S.; de Koning-Ward T. F. Genetic and chemical validation of Plasmodium falciparum aminopeptidase PfA-M17 as a drug target in the hemoglobin digestion pathway. Elife. 2022, 11, e80813 10.7554/eLife.80813. 36097817 PMC9470162

[ref18] Timm J.; Valente M.; García-Caballero D.; Wilson K. S.; González-Pacanowska D. Structural Characterization of Acidic M17 Leucine Aminopeptidases from the TriTryps and Evaluation of Their Role in Nutrient Starvation in Trypanosoma brucei. mSphere 2017, 2 (4), e00226-17 10.1128/mSphere.00226-17. 28815215 PMC5557676

[ref19] Morty R. E.; Morehead J. Cloning and characterization of a leucyl aminopeptidase from three pathogenic Leishmania species. J. Biol. Chem. 2002, 277 (29), 26057–26065. 10.1074/jbc.M202779200. 12006595

[ref20] Gu Y. Q.; Walling L. L. Identification of Residues Critical for Activity of the Wound-Induced Leucine Aminopeptidase (LAP-A) of Tomato. European Journal of Biochemistry/FEBS. 2002, 269, 1630–1640. 10.1046/j.1432-1327.2002.02795.x. 11895433

[ref21] Schneider P.; Glaser T. A. Characterisation of two soluble metalloexopeptidases in the protozoan parasite *Leishmania major*. Mol. Biochem. Parasitol. 1993, 62 (2), 223–231. 10.1016/0166-6851(93)90111-A. 8139615

[ref22] Ginger M. L.; Prescott M. C.; Reynolds D. G.; Chance M. L.; Goad L. J. Utilization of leucine and acetate as carbon sources for sterol and fatty acid biosynthesis by Old and New World Leishmania species, Endotrypanum monterogeii and Trypanosoma cruzi. Eur. J. Biochem. 2000, 267 (9), 2555–66. 10.1046/j.1432-1327.2000.01261.x. 10785375

[ref23] Aguado M. E.; González-Matos M.; Izquierdo M.; Quintana J.; Field M. C.; González-Bacerio J. Expression in *Escherichia coli*, purification and kinetic characterization of LAPLm, a Leishmania major M17-aminopeptidase. Protein Expr Purif. 2021, 183, 105877 10.1016/j.pep.2021.105877. 33775769

[ref24] Izquierdo M.; Lin D.; O’Neill S.; Zoltner M.; Webster L.; Hope A.; Gray D. W.; Field M. C.; González-Bacerio J. Development of a high-throughput screening assay to identify inhibitors of the major M17-leucyl aminopeptidase from *Trypanosoma cruzi* using RapidFire mass spectrometry. SLAS Discovery 2020, 25 (9), 1064–1071. 10.1177/2472555220923367. 32400260

[ref25] Izquierdo M.; Lin D.; O’Neill S.; Webster L. A.; Paterson C.; Thomas J.; Aguado M. E.; Colina Araújo E.; Alpízar-Pedraza D.; Joji H.; MacLean L.; Hope A.; Gray D. W.; Zoltner M.; Field M. C.; González-Bacerio J.; De Rycker M. Identification of a potent and selective LAPTc inhibitor by RapidFire-Mass Spectrometry, with antichagasic activity. PLoS Negl. Trop. Dis. 2024, 18 (2), e0011956 10.1371/journal.pntd.0011956. 38359089 PMC10901353

[ref26] Copeland R. A. Enzymes, A Practical Introduction to Structure, Mechanism, and Data Analysis, 2nd ed.; Wiley-VCH, Inc.: New York, 2000.

[ref27] Taylor M.; Ho J. MM/GBSA prediction of relative binding affinities of carbonic anhydrase inhibitors: effect of atomic charges and comparison with Autodock4Zn. J. Comput. Aided Mol. Des. 2023, 37 (4), 167–182. 10.1007/s10822-023-00499-0. 36930332 PMC10050039

[ref28] Corpas-Lopez V.; Wyllie S. Utilizing thermal proteome profiling to identify the molecular targets of anti-leishmanial compounds. STAR Protoc. 2021, 2 (3), 100704 10.1016/j.xpro.2021.100704. 34467225 PMC8384900

[ref29] Milne R.; Wiedemar N.; Corpas-Lopez V.; Moynihan E.; Wall R. J.; Dawson A.; Robinson D. A.; Shepherd S. M.; Smith R. J.; Hallyburton I.; Post J. M.; Dowers K.; Torrie L. S.; Gilbert I. H.; Baragaña B.; Patterson S.; Wyllie S. Toolkit of Approaches To Support Target-Focused Drug Discovery for Plasmodium falciparum Lysyl tRNA Synthetase. ACS Infect Dis. 2022, 8 (9), 1962–1974. 10.1021/acsinfecdis.2c00364. 36037410 PMC9469095

[ref30] Ambit A.; Woods K. L.; Cull B.; Coombs G. H.; Mottram J. C. Morphological events during the cell cycle of Leishmania major. Eukaryot Cell. 2011, 10 (11), 1429–1438. 10.1128/EC.05118-11. 21926331 PMC3209043

[ref31] Sajid M.; Robertson S. A.; Brinen L. S.; McKerrow J. H. Cruzain: the path from target validation to the clinic. Adv. Exp. Med. Biol. 2011, 712, 100–115. 10.1007/978-1-4419-8414-2_7. 21660661

[ref32] González-Bacerio J.; Varela A. C.; Aguado M. E.; Izquierdo M.; Méndez Y.; Del Rivero M. A.; Rivera D. G. Bacterial Metalo-Aminopeptidases as Targets in Human Infectious Diseases. Curr. Drug Targets. 2022, 23 (12), 1155–1190. 10.2174/1389450123666220316085859. 35297344

[ref33] Arastu-Kapur S.; Ponder E. L.; Fonović U. P.; Yeoh S.; Yuan F.; Fonović M.; Grainger M.; Phillips C. I.; Powers J. C.; Bogyo M. Identification of proteases that regulate erythrocyte rupture by the malaria parasite *Plasmodium falciparum*. Nat. Chem. Biol. 2008, 4 (3), 203–13. 10.1038/nchembio.70. 18246061

[ref34] Benz C.; Clucas C.; Mottram J. C.; Hammarton T. C. Cytokinesis in bloodstream stage *Trypanosoma brucei* requires a family of katanins and spastin. PLoS One 2012, 7 (1), e30367-12 10.1371/journal.pone.0030367. 22279588 PMC3261199

[ref35] Peña-Diaz P.; Vancová M.; Resl C.; Field M. C.; Lukeš J. A leucine aminopeptidase is involved in kinetoplast DNA segregation in Trypanosoma brucei. PLoS Pathog. 2017, 13 (4), e1006310 10.1371/journal.ppat.1006310. 28388690 PMC5397073

[ref36] Billington K.; Halliday C.; Madden R.; Dyer P.; Barker A. R.; Moreira-Leite F. F.; Carrington M.; Vaughan S.; Hertz-Fowler C.; Dean S.; Sunter J. D.; Wheeler R. J.; Gull K. Genome-wide subcellular protein map for the flagellate parasite *Trypanosoma brucei*. Nat. Microbiol. 2023, 8 (3), 533–547. 10.1038/s41564-022-01295-6. 36804636 PMC9981465

[ref37] Flipo M.; Beghyn T.; Leroux V.; Florent I.; Deprez B. P.; Deprez-Poulain R. F. Novel selective inhibitors of the zinc plasmodial aminopeptidase PfA-M1 as potential antimalarial agents. J. Med. Chem. 2007, 50 (6), 1322–1334. 10.1021/jm061169b. 17326615

[ref38] Drinkwater N.; Malcolm T. R.; McGowan S. M17 aminopeptidases diversify function by moderating their macromolecular assemblies and active site environment. Biochimie. 2019, 166, 38–51. 10.1016/j.biochi.2019.01.007. 30654132

[ref39] Machado P. A.; Carneiro M. P. D.; Sousa-Batista A. J.; Lopes F. J. P.; Lima A. P. C. A.; Chaves S. P.; Sodero A. C. R.; de Matos Guedes H. L. Leishmanicidal therapy targeted to parasite proteases. Life Sci. 2019, 219, 163–181. 10.1016/j.lfs.2019.01.015. 30641084

[ref40] Van Bocxlaer K.; Caridha D.; Black C.; Vesely B.; Leed S.; Sciotti R. J.; Wijnant G. J.; Yardley V.; Braillard S.; Mowbray C. E.; Ioset J. R.; Croft S. L. Novel benzoxaborole, nitroimidazole and aminopyrazoles with activity against experimental cutaneous leishmaniasis. Int. J. Parasitol Drugs Drug Resist. 2019, 11, 129–138. 10.1016/j.ijpddr.2019.02.002. 30922847 PMC6904836

[ref41] Wyllie S.; Brand S.; Thomas M.; De Rycker M.; Chung C. W.; Pena I.; Bingham R. P.; Bueren-Calabuig J. A.; Cantizani J.; Cebrian D.; Craggs P. D.; Ferguson L.; Goswami P.; Hobrath J.; Howe J.; Jeacock L.; Ko E. J.; Korczynska J.; MacLean L.; Manthri S.; Wyatt P. G.; et al. Preclinical candidate for the treatment of visceral leishmaniasis that acts through proteasome inhibition. Proc. Nat. Acad. Sci. U. S. A. 2019, 116 (19), 9318–9323. 10.1073/pnas.1820175116. PMC6511062 30962368

[ref42] Mowbray C. E.; Braillard S.; Glossop P. A.; Whitlock G. A.; Jacobs R. T.; Speake J.; Pandi B.; Nare B.; Maes L.; Yardley V.; Freund Y.; Wall R. J.; Carvalho S.; Bello D.; Van den Kerkhof M.; Caljon G.; Gilbert I. H.; Corpas-Lopez V.; Lukac I.; Patterson S.; Zuccotto F.; Wyllie S. DNDI-6148: A Novel Benzoxaborole Preclinical Candidate for the Treatment of Visceral Leishmaniasis. J. Med. Chem. 2021, 64 (21), 16159–16176. 10.1021/acs.jmedchem.1c01437. 34711050 PMC8591608

[ref43] Valiallahi A.; Vazifeh Z.; Gatabi Z. R.; Davoudi M.; Gatabi I. R. (2023). PLGA Nanoparticles as New Drug Delivery Systems in Leishmaniasis Chemotherapy: A Review of Current Practices. Current medicinal chemistry. 2023, 10.2174/0929867331666230823094737. 37612875

[ref44] Braillard S.; Keenan M.; Breese K. J.; Heppell J.; Abbott M.; Islam R.; Shackleford D. M.; Katneni K.; Crighton E.; Chen G.; Patil R.; Lee G.; White K. L.; Carvalho S.; Wall R. J.; Chemi G.; Zuccotto F.; González S.; Marco M.; Deakyne J.; Standing D.; Brunori G.; Lyon J. J.; Castañeda-Casado P.; Camino I.; Martinez Martinez M. S.; Zulfiqar B.; Avery V. M.; Feijens P. B.; Van Pelt N.; Matheeussen A.; Hendrickx S.; Maes L.; Caljon G.; Yardley V.; Wyllie S.; Charman S. A.; Chatelain E. DNDI-6174 is a preclinical candidate for visceral leishmaniasis that targets the cytochrome bc1. Sci. Transl Med. 2023, 15 (726), eadh9902 10.1126/scitranslmed.adh9902. 38091406 PMC7615677

[ref45] Kao C. C.; Wedes S. H.; Hsu J. W.; Bohren K. M.; Comhair S. A.; Jahoor F.; Erzurum S. C. Arginine metabolic endotypes in pulmonary arterial hypertension. Pulm Circ. 2015, 5 (1), 124–34. 10.1086/679720. 25992277 PMC4405713

[ref46] Mirdita M.; Steinegger M.; Söding J. MMseqs2 desktop and local web server app for fast, interactive sequence searches. Bioinformatics. 2019, 35 (16), 2856–2858. 10.1093/bioinformatics/bty1057. 30615063 PMC6691333

[ref47] Steinegger M.; Söding J. MMseqs2 enables sensitive protein sequence searching for the analysis of massive data sets. Nat. Biotechnol. 2017, 35 (11), 1026–1028. 10.1038/nbt.3988. 29035372

[ref48] Jumper J.; Evans R.; Pritzel A.; Green T.; Figurnov M.; Ronneberger O.; Tunyasuvunakool K.; Bates R.; Žídek A.; Potapenko A.; Bridgland A.; Meyer C.; Kohl S. A. A.; Ballard A. J.; Cowie A.; Romera-Paredes B.; Nikolov S.; Jain R.; Adler J.; Back T.; Bodenstein S.; Silver D.; Vinyals O.; Senior A. W.; Kavukcuoglu K.; Kohli P.; Hassabis D.; et al. Highly accurate protein structure prediction with AlphaFold. Nature. 2021, 596 (7873), 583–589. 10.1038/s41586-021-03819-2. 34265844 PMC8371605

[ref49] Fiser A.; Do R. K.; Sali A. Modeling of loops in protein structures. Protein Sci. 2000, 9 (9), 1753–1773. 10.1110/ps.9.9.1753. 11045621 PMC2144714

[ref50] Fiser A.; Sali A. ModLoop: automated modeling of loops in protein structures. Bioinformatics. 2003, 19 (18), 2500–2501. 10.1093/bioinformatics/btg362. 14668246

[ref51] Hanwell M. D.; Curtis D. E.; Lonie D. C.; Vandermeersch T.; Zurek E.; Hutchison G. R. Avogadro: an advanced semantic chemical editor, visualization, and analysis platform. J. Cheminform. 2012, 4 (1), 17 10.1186/1758-2946-4-17. 22889332 PMC3542060

[ref52] Santos-Martins D.; Forli S.; Ramos M. J.; Olson A. J. AutoDock4(Zn): an improved AutoDock force field for small-molecule docking to zinc metalloproteins. J. Chem. Inf Model. 2014, 54 (8), 2371–2379. 10.1021/ci500209e. 24931227 PMC4144784

[ref53] Valdés-Tresanco M. S.; Valdés-Tresanco M. E.; Valiente P. A.; Moreno E. AMDock: a versatile graphical tool for assisting molecular docking with Autodock Vina and Autodock4. Biol. Direct. 2020, 15 (1), 12 10.1186/s13062-020-00267-2. 32938494 PMC7493944

[ref54] Morris G. M.; Huey R.; Lindstrom W.; Sanner M. F.; Belew R. K.; Goodsell D. S.; Olson A. J. AutoDock4 and AutoDockTools4: Automated docking with selective receptor flexibility. J. Comput. Chem. 2009, 30 (16), 2785–2791. 10.1002/jcc.21256. 19399780 PMC2760638

[ref55] Abraham M. J.; Murtola T.; Schulz R.; Páll S.; Smith J. C.; Hess B.; Lindahl E. GROMACS: High performance molecular simulations through multi-level parallelism from laptops to supercomputers. SoftwareX 2015, 1-2, 19 10.1016/j.softx.2015.06.001.

[ref56] Maier J. A.; Martinez C.; Kasavajhala K.; Wickstrom L.; Hauser K. E.; Simmerling C. ff14SB: Improving the Accuracy of Protein Side Chain and Backbone Parameters from ff99SB. J. Chem. Theory Comput. 2015, 11 (8), 3696–3713. 10.1021/acs.jctc.5b00255. 26574453 PMC4821407

[ref57] He X.; Man V. H.; Yang W.; Lee T. S.; Wang J. A fast and high-quality charge model for the next generation general AMBER force field. J. Chem. Phys. 2020, 153 (11), 114502 10.1063/5.0019056. 32962378 PMC7728379

[ref58] Jakalian A.; Bush B. L.; Jack D. B.; Bayly C. I. Fast, efficient generation of high-quality atomic charges. AM1-BCC model: I. Method. J. Comput. Chem. 2000, 21, 132–146. 10.1002/(SICI)1096-987X(20000130)21:2<132::AID-JCC5>3.0.CO;2-P. 12395429

[ref59] Jakalian A.; Jack D. B.; Bayly C. I. Fast, efficient generation of high-quality atomic charges. AM1-BCC model: II. Parameterization and validation. J. Comput. Chem. 2002, 23 (16), 1623–1641. 10.1002/jcc.10128. 12395429

[ref60] Jorgensen W. L.; Chandrasekhar J.; Madura J. D.; Impey W. R.; Klein M. L. Comparison of simple potential functions for simulating liquid water. J. Chem. Phys. 1983, 79 (2), 926–935. 10.1063/1.445869.

[ref61] Yang W.; Riley B. T.; Lei X.; Porebski B. T.; Kass I.; Buckle A. M.; McGowan S. Generation of AMBER force field parameters for zinc centres of M1 and M17 family aminopeptidases. J. Biomol Struct Dyn. 2018, 36 (10), 2595–2604. 10.1080/07391102.2017.1364669. 28782419

[ref62] Hess B. P-LINCS: A Parallel Linear Constraint Solver for Molecular Simulation. J. Chem. Theory Comput. 2008, 4 (1), 116–122. 10.1021/ct700200b. 26619985

[ref63] Miyamoto S.; Kollman P. A. Settle: An analytical version of the SHAKE and RATTLE algorithm for rigid water models. J. Comput. Chem. 1992, 13, 952–962. 10.1002/jcc.540130805.

[ref64] Nguyen H.; Roe D. R.; Simmerling C. Improved Generalized Born Solvent Model Parameters for Protein Simulations. J. Chem. Theory Comput. 2013, 9 (4), 2020–2034. 10.1021/ct3010485. 25788871 PMC4361090

[ref65] Valdés-Tresanco M. E.; Valdés-Tresanco M. S.; Moreno E.; Valiente P. A. Assessment of Different Parameters on the Accuracy of Computational Alanine Scanning of Protein-Protein Complexes with the Molecular Mechanics/Generalized Born Surface Area Method. J. Phys. Chem. B 2023, 127 (4), 944–954. 10.1021/acs.jpcb.2c07079. 36661180

[ref66] Park E. K.; Jung H. S.; Yang H. I.; Yoo M. C.; Kim C.; Kim K. S. Optimized THP-1 differentiation is required for the detection of responses to weak stimuli. Inflamm Res. 2007, 56 (1), 45–50. 10.1007/s00011-007-6115-5. 17334670

[ref67] Daigneault M.; Preston J. A.; Marriott H. M.; Whyte M. K.; Dockrell D. H. The identification of markers of macrophage differentiation in PMA-stimulated THP-1 cells and monocyte-derived macrophages. PLoS One. 2010, 5 (1), e8668 10.1371/journal.pone.0008668. 20084270 PMC2800192

[ref68] Paradela L. S.; Wall R. J.; Carvalho S.; Chemi G.; Corpas-Lopez V.; Moynihan E.; Bello D.; Patterson S.; Güther M. L. S.; Fairlamb A. H.; Ferguson M. A. J.; Zuccotto F.; Martin J.; Gilbert I. H.; Wyllie S. Multiple unbiased approaches identify oxidosqualene cyclase as the molecular target of a promising anti-leishmanial. Cell Chem. Biol. 2021, 28 (5), 711–721. 10.1016/j.chembiol.2021.02.008. 33691122 PMC8153249

[ref69] Perez-Riverol Y.; Csordas A.; Bai J.; Bernal-Llinares M.; Hewapathirana S.; Kundu D. J.; Inuganti A.; Griss J.; Mayer G.; Eisenacher M.; Pérez E.; Uszkoreit J.; Pfeuffer J.; Sachsenberg T.; Yilmaz S.; Tiwary S.; Cox J.; Audain E.; Walzer M.; Jarnuczak A. F.; Ternent T.; Brazma A.; Vizcaíno J. A. The PRIDE database and related tools and resources in 2019: improving support for quantification data. Nucleic Acids Res. 2019, 47 (D1), D442–D450. 10.1093/nar/gky1106. 30395289 PMC6323896

[ref70] Tyanova S.; Temu T.; Sinitcyn P.; Carlson A.; Hein M. Y.; Geiger T.; Mann M.; Cox J. The Perseus computational platform for comprehensive analysis of (prote)omics data. Nat. Methods. 2016, 13 (9), 731–40. 10.1038/nmeth.3901. 27348712

[ref71] Allan C.; Burel J. M.; Moore J.; Blackburn C.; Linkert M.; Loynton S.; Macdonald D.; Moore W. J.; Neves C.; Patterson A.; Porter M.; Tarkowska A.; Loranger B.; Avondo J.; Lagerstedt I.; Lianas L.; Leo S.; Hands K.; Hay R. T.; Patwardhan A.; Best C.; Kleywegt G. J.; Zanetti G.; Swedlow J. R. OMERO: flexible, model-driven data management for experimental biology. Nat. Methods. 2012, 9 (3), 245–53. 10.1038/nmeth.1896. 22373911 PMC3437820

