# Supplementary material for: Identification and Validation of Compounds Targeting Leishmania major Leucyl-Aminopeptidase M17
Source: ACS Infect Dis. 2024 May 16;10(6):2002–17. doi: 10.1021/acsinfecdis.4c00009 (PMC11184559; doi:10.1021/acsinfecdis.4c00009)
Supplement: Supplementary file 1 — id4c00009_si_001.pdf [file id4c00009_si_001.pdf]

“Supporting Information”

Identification and validation of compounds targeting *Leishmania major* leucyl-aminopeptidase M17

Mirtha E Aguado[1], Sandra Carvalho[2], Mario E Valdés-Tresanco[3], De Lin[2], Norma Padilla-Mejia[2], Victoriano Corpas-Lopez[2], Martina Tesařová[4], Julius Lukeš[4,5], David Gray[2], Jorge González-Bacerio[1]*, Susan Wyllie[2]* and Mark C. Field[2,4]*

[1]Center for Protein Studies, Faculty of Biology, University of Havana, 10400 Havana, [2]Wellcome Centre for Anti-Infective Research, School of Life Sciences, University of Dundee, DD1 4HN Scotland, UK, [3]Centre for Molecular Simulations, University of Calgary, Calgary AB T2N 1N4, Canada, [4]Institute of Parasitology, Biology Centre, Czech Academy of Sciences, 37005 České Budějovice, Czech Republic and [5]Faculty of Sciences, University of South Bohemia, 37005 České Budějovice, Czech Republic.

***Corresponding authors:** JGB; jogoba@fbio.uh.cu, SW; swyllie@dundee.ac.uk and MCF; mfield@mac.com, mcfield@dundee.ac.uk.

Table S1: Z´ robustness coefficients and signal-to-noise ratios of mock screen for r*Lm*LAP

Plate	Z´ robustness coefficient	S/B
1	0.82	14.9
2	0.89	16.2

A mock screen was performed with RapidFire-MS (see methods). Recombinant *Lm*LAP enzyme was tested at 5 nM and 10 μM LSTVIVR peptide substrate for a 60 min reaction time. S/B: Signal-to-noise ratio.

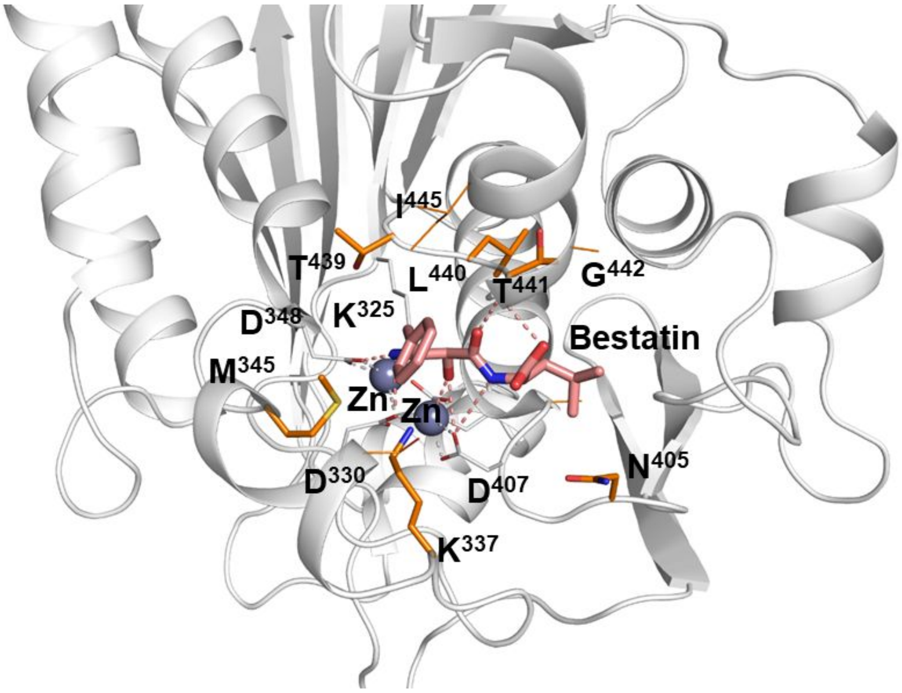

Figure S1: Bioinformatics analyses of r*Lm*LAP:bestatin interactions using PyMol. Predicted binding mode of the r*Lm*LAP:bestatin complex. For clarity, the interacting residues within 4Å of the inhibitor are represented as lines and van der Waals interactions between inhibitor and interacting residues are not indicated. Color codes: Inhibitor atoms: Carbon in salmon, oxygen in red, and nitrogen in blue; LAP residues interacting with Zn^{2+} ions: carbon in gray, oxygen in red, and nitrogen in blue; LAP's inhibitor interacting residues: carbon in orange, oxygen in red, nitrogen in blue and sulphur in yellow. The Zn^{2+} ions are represented as gray spheres. Zn^{2+} coordination is represented by dotted lines.

Bestatin binding pose is similar that of described for bestatin with other related aminopeptidases (e.g. *Trypanosoma brucei* acidic LAP; PDB: 5NTD; Timm *et al*., 2017).

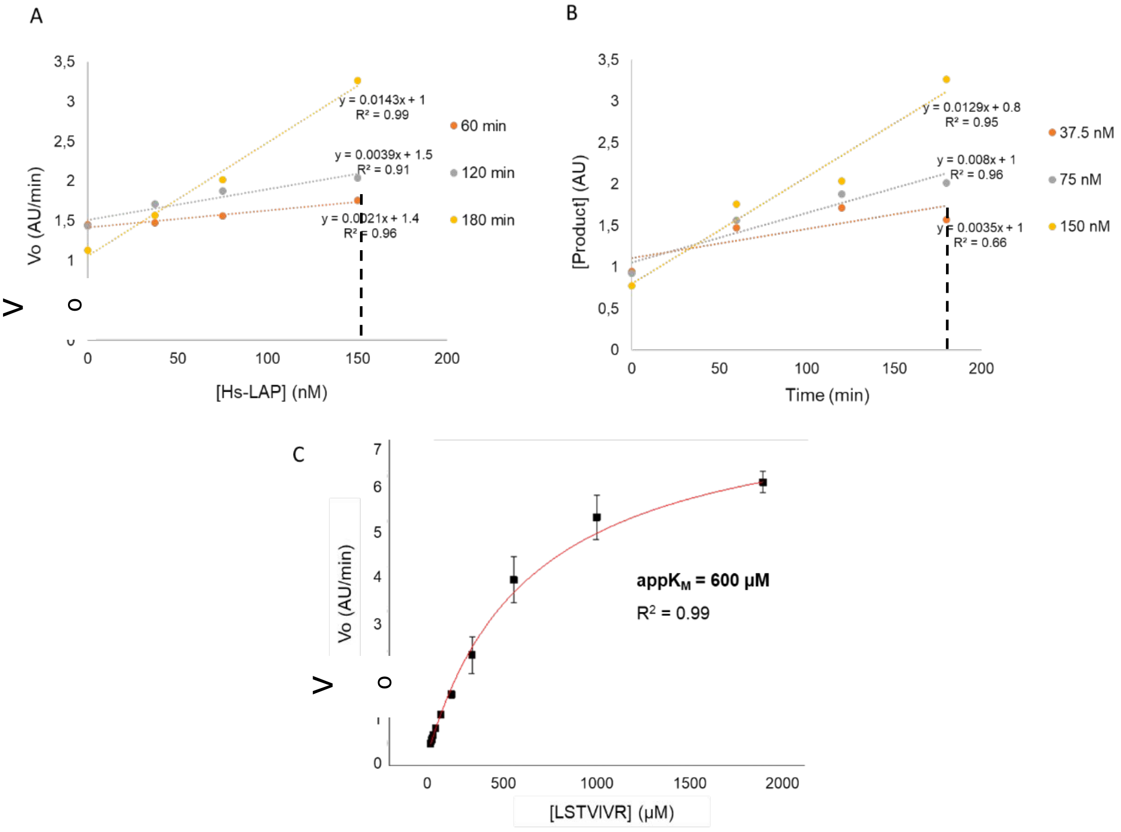

Figure S2: Optimization of RapidFire-MS for *Hs*LAP. (A) Relationship between v_0 and enzyme concentration at 60, 120 and 180min reaction times. The selected enzyme concentration (150 nM) is shown with a dashed line. (B) Typical curves at 37, 75 and 150 nM enzyme for a 180 min reaction time. The selected reaction time (180 min) is indicated by a dashed line. In both experiments 2 mM (3.33 × $appK_M$) LSTVIVR peptide substrate was used. Straight line fit equations and coefficients for determination of the linear fits (R^2) are shown. (C) Determination of $appK_M$ for *Hs*LAP and LSTVIVR peptide substrate. Enzyme was assayed at 150 nM towards ten LSTVIVR peptide substrate concentrations, prepared by serial dilution in water between 3.9 and 2000 μM. The reaction time was 180 min. All assays were performed with 1 mM $ZnCl_2$. A Michaelis-Menten rectangular hyperbola function was fitted to the experimental data using OriginPro8 SR0 software. R^2: coefficient of

determination for the rectangular hyperbola fitting. Experimental data are presented as mean ± standard deviations (n = 3). In (A) and (B), standard deviations are included in the 10% of the mean. v_0: initial reaction velocity. AU: arbitrary units. appK_M: apparent Michaelis-Menten constant.

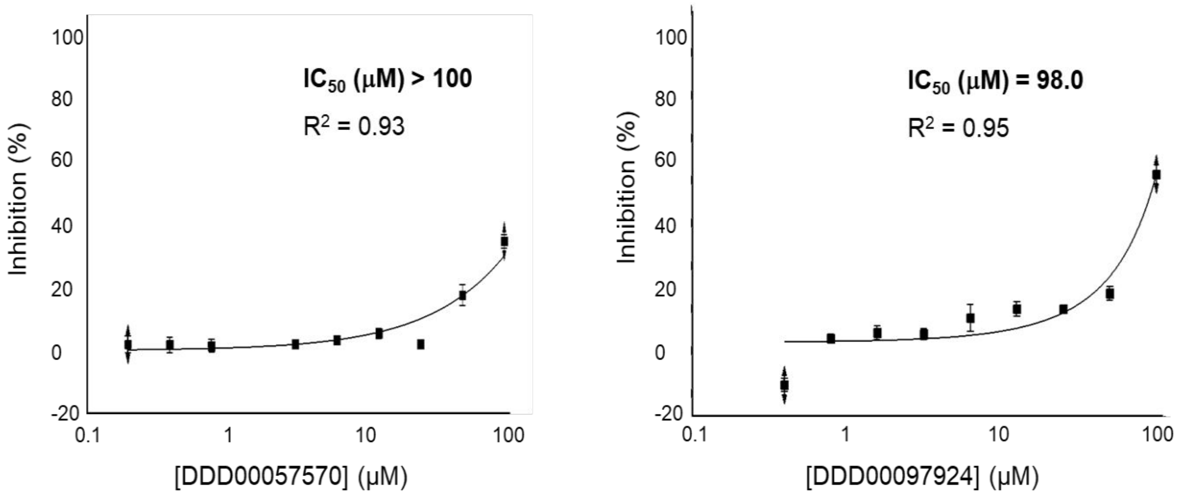

Figure S3: Concentration response for inhibition of *Hs*LAP by DDD00057570 and DDD00097924. *Hs*LAP was assayed at 150 nM with 600 μM LSTVIVR peptide substrate, 1 mM $ZnCl_2$ and 180 min reaction time by RapidFire-MS. IC_{50} was calculated by non-linear fit of the dose-response to experimental data using OriginPro8 SR0 software. R^2: Determination coefficient. Experimental data are presented as mean ± standard deviations (n = 3).

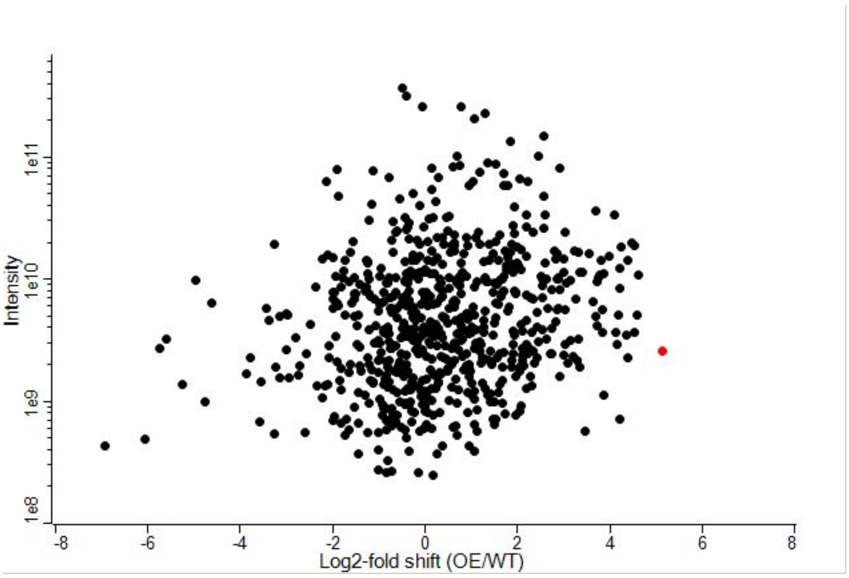

Figure S4: Confirmation of *Lm*LAP overexpression in transgenic promastigotes by TMT quantitation. Relative levels of proteins in wild-type (WT) versus transgenic overexpressing (OE) cell lines were directly compared. *Lm*LAP is highlighted in red.

Table S2: Impact of *Lm*LAP overexpression on drug sensitivity.

**ID**	**EC_{50} value ± SD, µM**	
	WT	*LAP-OE*
DDD00057570	1.8 ± 0.2	7 ± 1
DDD00097924	3 ± 0.2	11 ± 1

Data represents the weighted mean ± SD (n = 5).

Table S3: iTPP hits stabilized in the presence of DDD00057570.

Enrichment (log_2) replica A	Enrichment (log_2) replica B	Peptides identified	Gene ID	Function
1.47	1.34	9	LmjF.11.0630	metallo-peptidase, Clan MF, Family M17
1.38	1.17	7	LmjF.33.2570	metallo-peptidase, Clan MF, Family M17
1.36	1.64	5	LmjF.35.1670	60S ribosomal protein L26, putative

Cut-off value log_2 enrichment > 1 in both replicates A and B

Table S4: iTPP hits stabilized in the presence of DDD00097924.

Enrichment (log_2) replica A	Enrichment (log_2) replica B	Peptides identified	Gene ID	Function
2.19	1.99	9	LmjF.11.0630	metallo-peptidase, Clan MF, Family M17
1.97	1.76	7	LmjF.33.2570	metallo-peptidase, Clan MF, Family M17
1.71	1.97	5	LmjF.35.1670	60S ribosomal protein L26, putative
1.31	1.21	7	LmjF.23.0040	peroxidoxin

Cut-off value log_2 enrichment > 1 in both replicates A and B

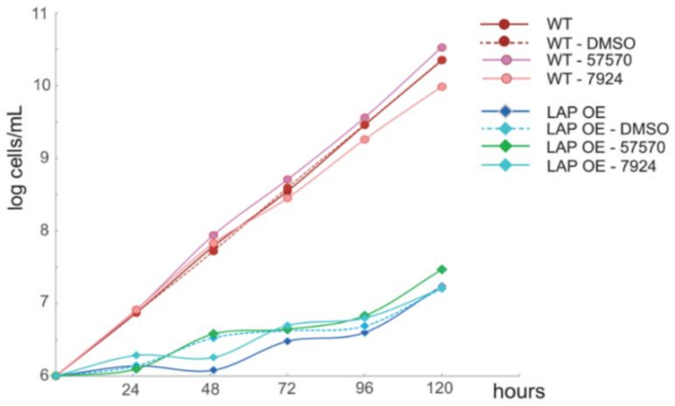

Figure S5: Cumulative growth of wild-type (WT) and *Lm*LAP overexpressing (OE) parasites. Parasites were grown in the presence of

DDD00057570 and DDD00097924 at the corresponding EC_{50} and cell density counted every 24 h.

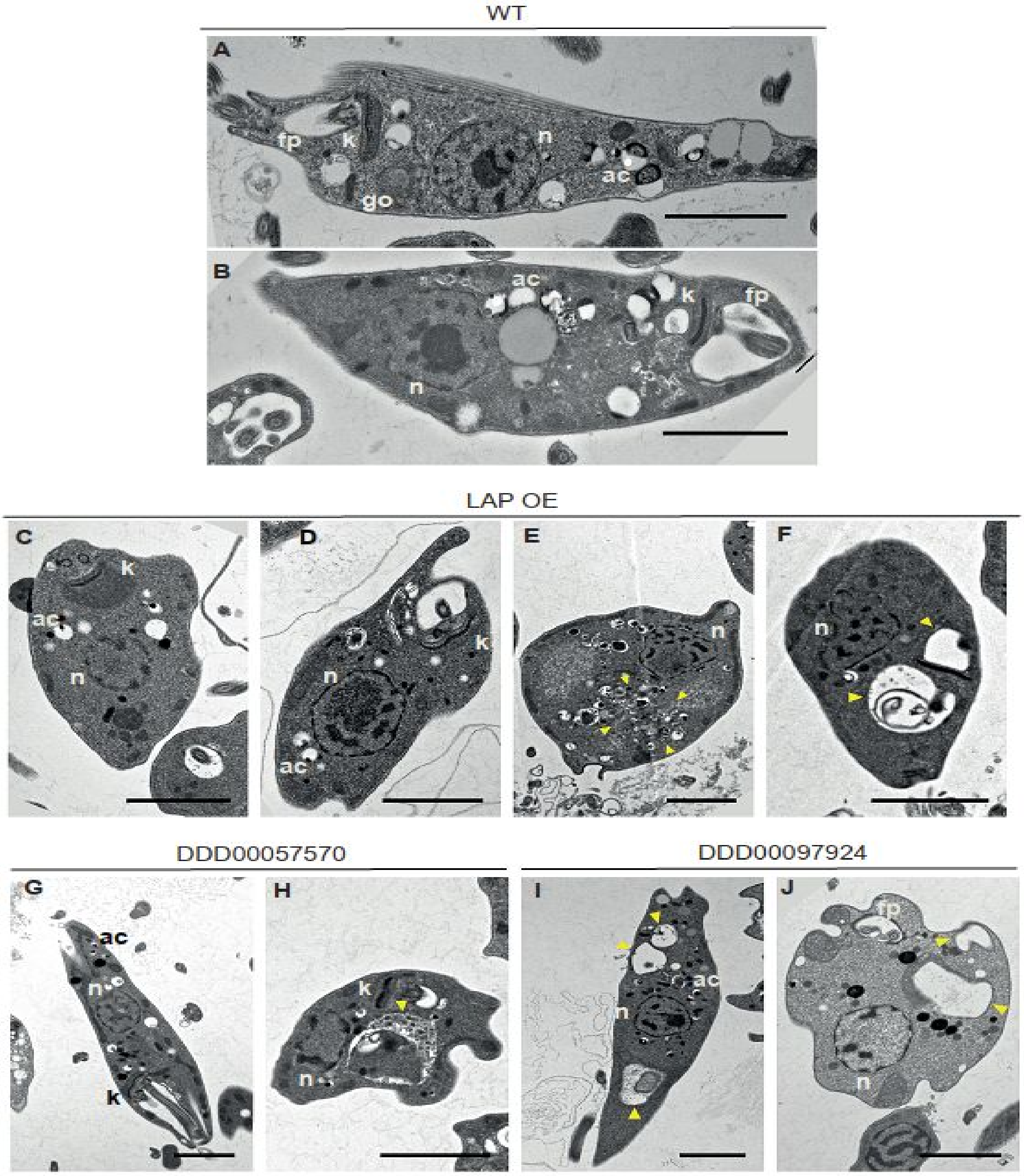

Figure S6: Ultrastructure morphology of *L. major* promastigotes after treatment with compounds DDD00057570 and DDD00097924. (A-B) Wild-type (WT) cells without any treatment. Notice the elongated cellular form. (C-F) Cells overexpressing LAP (OE). A combination of normal (panel C and D) and abnormal (panel E and F) morphologies were observed, including swollen kinetoplast (C), swollen cell morphology (E) and the presence of vesicle-like

compartments (E, F; yellow arrowheads). (G-H) Cells treated with compound DDD00057570. (I-J) Cells treated with compound DDD00097924. Both compounds partially restore the elongated cell shape in the population, likely reversing the toxic effects of the overexpression of LAP (G, I). However, a rounded cell shape and the occurrence of aberrant vesicle-like compartments remain (H, J). Membrane-bounded compartments are present in different morphologies and sizes. Aggregation of small compartments (E, H) together with the presence of extra-large compartments (F, J) can be observed. ac, acidocalcisome; fp, flagellar pocket; k, kinetoplast; n, nucleus. Bar scale 2 μm.

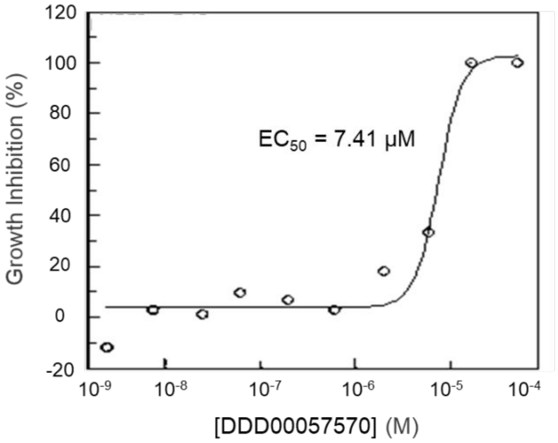

Inhibitor ID	EC_{50} values, (μM)			CSI (RAW 264.7)	CSI (THP-1)
	L. donovani	RAW 264.7	THP-1		
DDD00057570	7	102	>100	14	>14

Figure S7: EC_{50} values for DDD00057570 against *Leishmania donovani* intracellular amastigotes. For determination of anti-leishmanial activity parasites were exposed to compounds for 96 h, after which parasite and host cells were stained with 5 μg/mL Hoechst 33342 dye, and parasite numbers measured by imaging. For cytotoxicity, EC_{50} values were determined by exposing macrophages to test compounds for 48 h, with MTT assays used to determine cell viability. Data are presented as mean of three replicates. Standard deviations are within 10% of the mean values. EC_{50}: half-maximum effective concentration. CSI: cellular selectivity index (EC_{50} macrophage / EC_{50} *L. donovani*).
